# An Injectable Epigenetic Autophagic Modulatory Hydrogel for Boosting Umbilical Cord Blood NK Cell Therapy Prevents Postsurgical Relapse of Triple‐Negative Breast Cancer

**DOI:** 10.1002/advs.202201271

**Published:** 2022-06-16

**Authors:** Yihang Gong, Wenjie Chen, Xiuxing Chen, Yizhan He, Hua Jiang, Xijian Zhang, Lijie Pan, Beibei Ni, Fan Yang, Yan Xu, Qi Zhang, Lei Zhou, Yusheng Cheng

**Affiliations:** ^1^ Department of Hepatic Surgery and Liver Transplantation Center & Guangdong Provincial Key Laboratory of Liver Disease Research The Third Affiliated Hospital Sun Yat‐sen University Guangzhou 510630 China; ^2^ Biotherapy Centre & Cell‐Gene Therapy Translational Medicine Research Centre The Third Affiliated Hospital Sun Yat‐sen University Guangzhou 510630 China; ^3^ Guangdong Provincial Key Laboratory of Malignant Tumor Epigenetics and Gene Regulation Department of Medical Oncology Sun Yat‐sen Memorial Hospital Sun Yat‐sen University Guangzhou 510120 China; ^4^ Department of Breast & Thyroid Surgery The Third Affiliated Hospital Sun Yat‐sen University Guangzhou 510630 China; ^5^ Guangzhou Key Laboratory of Spine Disease Prevention and Treatment Department of Spine Surgery The Third Affiliated Hospital Guangzhou Medical University Guangzhou 510150 China

**Keywords:** hydrogel, sustained drug release, TNBC, UCB‐NK cell therapy, wound healing

## Abstract

Triple‐negative breast cancer (TNBC) exhibits resistance to conventional treatments due to the presence of cancer stem cells (CSCs), causing postsurgical relapse and a dismal prognosis. Umbilical cord blood natural killer (UCB‐NK) cell‐based immunotherapy represents a promising strategy for cancer treatment. However, its therapeutic efficacy is greatly restrained by downregulation of the NK cell activation ligand MHC class I‐related chain A/B (MICA/B) and autophagy‐mediated degradation of NK cell‐derived granzyme B (GZMB) in CSCs. Herein, it is demonstrated that suberoylanilide hydroxamic acid (SAHA) epigenetically downregulates let‐7e‐5p and miR‐615‐3p to increase MICA/B expression and that 3‐methyl adenine (3MA) inhibits autophagy‐mediated GZMB degradation, thereby sensitizing breast CSCs to UCB‐NK cells. Then, an injectable hydrogel is designed to codeliver SAHA and 3MA to enhance UCB‐NK cell infusion efficacy in TNBC. The hydrogel precursors can be smoothly injected into the tumor resection bed and form a stable gel in situ, allowing for a pH‐sensitive sustained release of SAHA and 3MA. Moreover, UCB‐NK cell infusion in combination with the hydrogel efficiently controls postsurgical relapse of TNBC. In addition, the hydrogel exhibits good hemostasis and wound‐healing functions. Therefore, the work provides proof of concept that an injectable epigenetic autophagic modulatory hydrogel augments UCB‐NK cell therapy to combat postsurgical relapse of TNBC.

## Graphical abstract

An injectable hydrogel for restoring major histocompatibility complex class I‐related chain A/B (MICA/B) expression and mitigating autophagic flux in breast cancer stem cells (BCSCs) is developed to enhance the efficacy of umbilical cord blood natural killer (UCB‐NK) cell infusion in combating postsurgical triple‐negative breast cancer (TNBC) relapse.

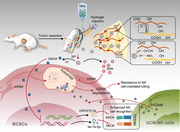

## 1 Introduction

Breast cancer (BC) imposes a huge economic and health burden on women worldwide.^[^ ^1^ ^]^ Triple‐negative breast cancer (TNBC), which lacks expression of the estrogen receptor (ER), human epidermal growth factor receptor 2 (HER2), and progesterone receptor (PR), is a highly aggressive type of BC, accounting for approximately 15% of all BC cases.^[^ ^2^ ^]^ Tumor resection in combination with chemotherapy is currently recommended for TNBC treatment. However, it is rare for TNBC patients to have long‐term survival due to tumor relapse after treatment.^[^ ^3^, ^4^ ^]^ There is mounting evidence that a unique subpopulation of cancer cells exists in TNBC, referred to as breast cancer stem cells (BCSCs), which can be identified by aldehyde dehydrogenase 1 (ALDH1).^[^ ^4^, ^5^ ^]^ BCSCs exhibit resistance or no response to conventional chemotherapeutic agents, radiation, and ER‐, HER2‐ or PR‐targeted drugs, consequently resulting in tumor relapse.^[^ ^6^ ^]^ Hence, developing efficient strategies to eradicate BCSCs is an unmet demand for TNBC treatment.

Natural killer (NK) cells, one of the most crucial innate immune cells for immunosurveillance, display rapid and potent antitumor immunity.^[^ ^7^ ^]^ Clinical studies have confirmed a converse correlation between NK cell activation and tumor progression in breast cancer,^[^ ^8^ ^]^ hepatocellular carcinoma,^[^ ^9^ ^]^ and gastric cancer.^[^ ^10^ ^]^ Moreover, there is preclinical evidence that adoptive NK cell therapy shows promise as an alternative strategy for a broad spectrum of malignancies, including breast cancer.^[^ ^11^, ^12^, ^13^ ^]^ Compared to other immunotherapeutic approaches, adoptive NK cell therapy has several unique merits, such as independence from major histocompatibility complex (MHC)‐antigen restriction, minimal adverse effects, and better feasibility for “off‐the‐shelf” manufacturing.^[^ ^14^ ^]^ For these reasons, hundreds of clinical trials exploring adoptive NK cell therapy safety and efficacy in hematological malignancies and solid tumors have been launched and are in full swing (https://www.clinicaltrials.gov). The NK cells used in those clinical trials were derived from multiple sources, including autologous peripheral blood (PB) NK cells, allogeneic PB NK cells, umbilical cord blood NK (UCB‐NK) cells, and NK cell lines. Among these sources, UCB is a very attractive choice since it is a relatively exhaustless and easily accessible source, and UCB‐NK cells have a higher proliferative capacity, stronger cytotoxicity, faster immune reconstitution, less stringent requirement for HLA matching, and reduced risk of graft versus host disease than their counterparts from other sources.^[^ ^15^, ^16^ ^]^ In particular, UCB‐NK cells have been revealed to exhibit robust cytotoxicity to TNBC cells,^[^ ^17^ ^]^ and NK cells can recognize and selectively attack cancer stem cells.^[^ ^18^ ^]^ Based on this knowledge, we postulated that UCB‐NK cell infusion may represent a promising alternative strategy for TNBC treatment.

Despite these promising findings, the efficacy of adoptive NK cell therapy is dramatically limited by immune evasion resulting from the reduced expression of NK cell activation ligand MHC class I‐related chain A and B (MICA/B) in CSCs.^[^ ^19^, ^20^ ^]^ Therefore, it is of great significance to develop combination strategies for restoring MICA/B expression in BCSCs to augment NK‐cell antitumor immunity. Suberoylanilide hydroxamic acid (SAHA), an epigenetic drug approved by the US Food and Drug Administration (FDA) for cancer treatment, was found to elevate MICA/B expression via epigenetic downregulation of the miR‐17‐92 cluster in hepatocellular carcinoma cells.^[^ ^21^ ^]^ Similarly, herein we demonstrated for the first time that SAHA significantly increased MICA/B expression in BCSCs by repressing let‐7e‐5p and miR‐615‐3p, implying the potential of SAHA as a candidate drug to improve UCB‐NK cell infusion efficacy in TNBC. Autophagy is a lysosomal degradation process in cells, which has been shown to be aberrantly upregulated in BC cells, especially BCSCs.^[^ ^4^ ^]^ Of note, autophagy in cancer cells can degrade NK cell‐derived granzyme B (GZMB), consequently limiting NK cell effector function.^[^ ^22^ ^]^ Consistently, in this study, we also found that the combination of SAHA and the autophagy inhibitor 3‐methyl adenine (3MA) performed much better in enhancing NK cell cytotoxicity to BCSCs than single exposure to SAHA or 3MA. Therefore, UCB‐NK cell infusion with coadministration of SAHA and 3MA could be a more optimized immunotherapeutic strategy for TNBC.

**Figure 1:**
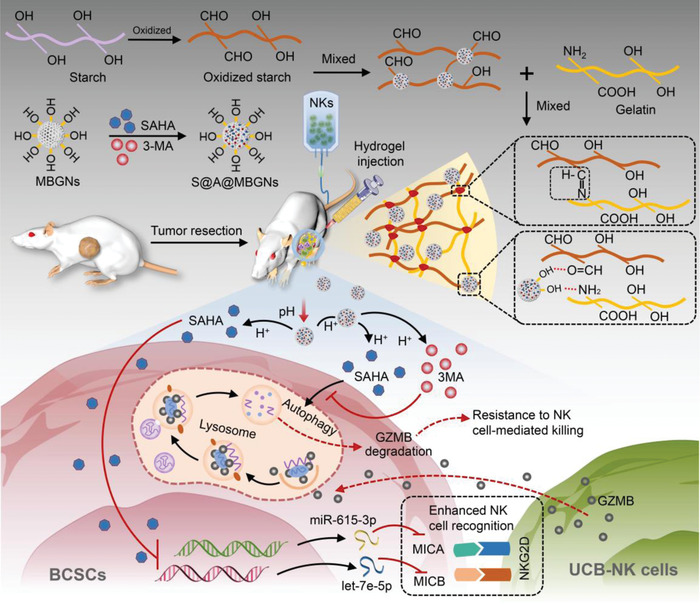
Schematic of the preparation of GOSAM and the combined therapy of GOSAM and UCB‐NK cell infusion for TNBC. The precursor of Gel‐OS/SAHA@3MA@MBGNs hydrogel (GOSAM) was injected into TNBC resection wounds and formed a gel in situ. The in situ‐formed hydrogel elevated MICA/B expression via epigenetic downregulation of the miR‐615‐3p and let‐7e‐5p cluster and inhibited autophagy to prevent the degradation of granzyme B, which improved the therapeutic efficacy of UCB‐NK cell infusion.

Epigenetic modifications are reversible, and autophagy can be rapidly induced in response to relevant stimulation.^[^ ^23^, ^24^ ^]^ A sustained supply of epigenetic drugs and autophagy inhibitors is thus warranted for treating human diseases. Hydrogels have been vastly explored as innovative drug carriers for solid cancer therapy and can provide local delivery of agents with high bioavailability.^[^ ^25^, ^26^ ^]^ Importantly, several traits of the tumor microenvironment (TME), such as its acidic pH, can be utilized to develop bioresponsive hydrogels to sustain controlled drug release, thereby reducing doses and times of administration.^[^ ^27^, ^28^ ^]^ In addition, hydrogels can be designed to possess hemostatic and wound healing properties,^[^ ^29^, ^30^, ^31^ ^]^ which may accelerate patient postoperative recovery and even helps to reduce tumor relapse.^[^ ^32^ ^]^ These merits make hydrogel‐based platforms attractive drug delivery systems for cancer treatment. Herein, we developed an in situ injectable pH‐responsive hydrogel encapsulating SAHA and 3MA to boost UCB‐NK cell infusion efficacy in TNBC (**Figure** **1**). In our previous study,^[^ ^33^ ^]^ we reported biocompatible mesoporous bioactive glass nanoparticles (MBGNs), which have a high surface ratio, indicating their potential as drug nanocarriers. Hence, we first loaded SAHA and 3MA into MBGNs to prepare SAHA@3MA@MBGNs. Next, we incorporated SAHA@3MA@MBGNs into oxidized starch (OS) and then mixed OS with gelatin (Gel) to fabricate the polymer hydrogel, hereafter referred to as the Gel‐OS/SAHA@3MA@MBGN hydrogel (GOSAM). There are at least two mechanisms for the gelation of GOSAM. The aldehyde groups in OS and the amino groups in Gel can react to generate a Schiff's base bond to meditate the chemical crosslinking in the polymer.^[^ ^33^ ^]^ Besides, an abundance of silanol groups (Si‐OH) can form on the outer surfaces of MBGNs in an aqueous environment due to the hydration reaction, which interacts with the amino groups to consolidate the crosslinking in the polymer hydrogel.^[^ ^34^ ^]^ The GOSAM precursor can be smoothly injected into the tumor resection bed, forming a biocompatible and biodegradable gel in situ. GOSAM allows for a pH‐responsive sustained release of SAHA and 3MA, which restores MICA/B expression and inhibits autophagy in BCSCs. Local injection of GOSAM greatly improves UCB‐NK cell infusion to control TNBC growth and postresection relapse. Meanwhile, GOSAM has good adhesive hemostatic performance and wound‐healing capacity. Therefore, our work provides proof of concept that an injectable epigenetic autophagic modulatory hydrogel with hemostasis and wound healing functions enhances adoptive UCB‐NK cell therapy to combat postresection relapse of TNBC.

## 2 Results and Discussion

### 2.1 Isolation and Identification of Breast Cancer Stem Cells (CSCs)

**Figure 2:**
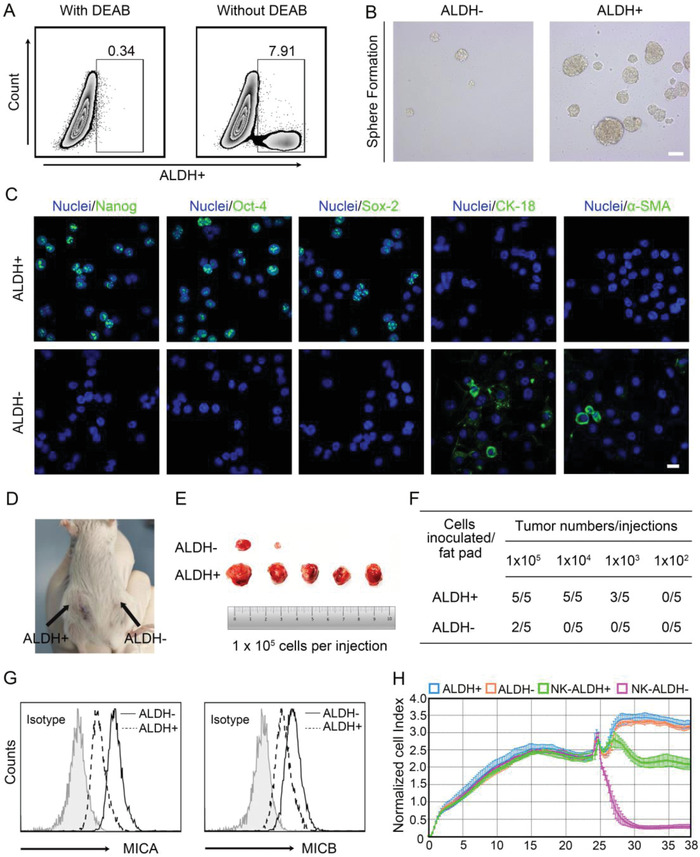
Isolation and characterization of stem‐like properties of BCSCs. A) Isolation of ALDH^+^ and ALDH‐ cell subsets by flow cytometry. B) Typical mammosphere formation by ALDH^−^ and ALDH^+^ cells in stem cell medium, scale bar = 200 µm. C) Immunofluorescence staining of Nanog, Oct‐4, Sox‐2, CK‐18, and *α*‐SMA in ALDH^+^ and ALDH^−^ cells, scale bar = 20 µm. D,E) Representative image of subcutaneous tumors generated by ALDH^+^ and ALDH^−^ cells (1 × 10^5^ cells per injection) in NSG mice. F) The tumorigenic incidence by injection of different numbers of tumor cells in mice, *n*  =  5. G) Relative expression of MICA/B in ALDH^+^ and ALDH^−^ cell subsets. H) The lysis effect of UCB‐NK cells on tumor cells was assessed using a cell dynamic detector. Data are presented as the mean ± SD, (*n*  =  3). *p < 0.05, **p < 0.01, and ***p < 0.001.

TNBC has a rather high risk of postresection relapse and distant metastasis, severely affecting the long‐term survival of patients.^[^ ^3^, ^4^ ^]^ Enrichment of breast cancer stem cells (BCSCs) in residual tumors has been revealed to be a crucial driver of postresection relapse of TNBC.^[^ ^4^, ^5^ ^]^ The development of efficient strategies to eradicate BCSCs is thus an unmet clinical demand for TNBC therapy. Recently, umbilical cord blood NK (UCB‐NK) cell infusion has attracted great interest as an alternative strategy for cancer treatment.^[^ ^18^ ^]^ However, CSCs often have a reduced expression of ligands for NK cell activation, MHC class I‐related chain A and B (MICA/B), which helps them escape from NK cell killing.^[^ ^19^, ^20^ ^]^ Therefore, it is of great importance to develop combination strategies for restoring MICA/B expression in BCSCs to improve NK cell antitumor immunity. Dysregulation of histone acetylation is a key mechanism for reduced MICA/B expression in CSCs, which implied that modulation of histone acetylation may hold promise for upregulating MICA/B expression. Suberoylanilide hydroxamic acid (SAHA) is an epigenetic drug approved by the US Food and Drug Administration (FDA) for cancer therapy.^[^ ^35^ ^]^ Importantly, SAHA has been demonstrated to increase MICA/B levels in hepatocellular carcinoma cells.^[^ ^21^ ^]^ Therefore, we investigated whether SAHA could serve as a candidate drug for restoring MICA/B expression in BCSCs. To address this issue, we first isolated ALDH1^+^ cells from a human TNBC cell line (MDA‐MB‐231) (**Figure** **2**A), which has been proposed to be BCSCs,^[^ ^4^, ^5^ ^]^ for subsequent experiments. As expected, we demonstrated that ALDH1^+^ MDA‐MB‐231 cells displayed potent self‐renewal potential, highly expressed stem cell markers, and promoted tumorigenicity in vivo (Figure 2B–F and Figure S1A, Supporting Information). In addition, ALDH1+ BC cells exhibited much lower expression of MICA/B and less sensitivity to NK cell‐mediated lysis than ALDH1‐ BC cells (Figure 2G,H and Figure S1B, Supporting Information). These results verified that ALDH1^+^ MDA‐MB‐231 cells exhibit the typical properties of CSCs.

### 2.2 SAHA Upregulates MICA/B Expression by Inhibiting miR‐615‐3p and let‐7e‐5p in BCSCs

**Figure 3:**
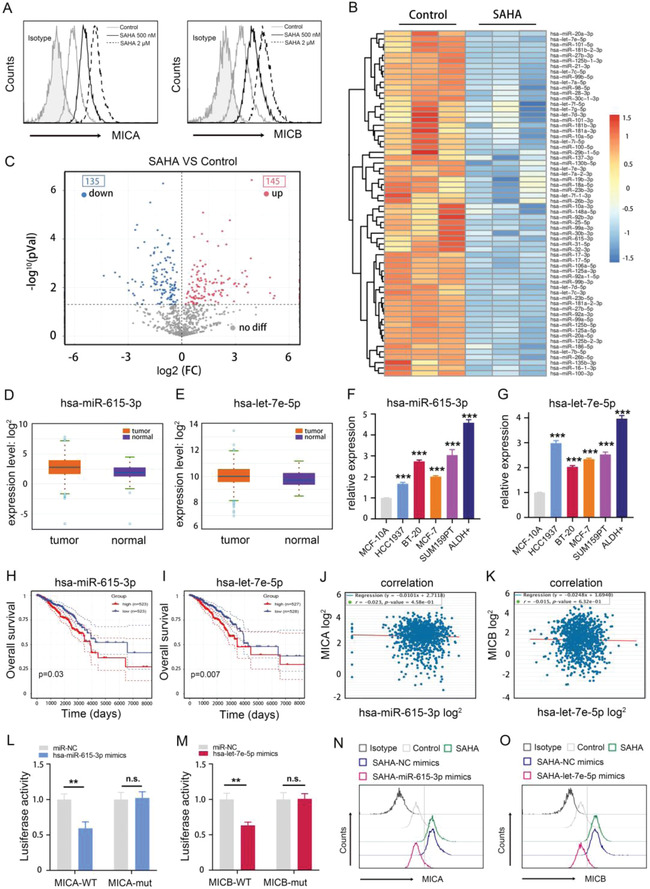
SAHA elevates MICA/B expression via epigenetic downregulation of miR‐615‐3p and the let‐7e‐5p cluster. A) The effect of different concentrations of SAHA on the expression of MICA/B in ALDH+ BCs was analyzed by flow cytometry. B,C) The effect of SAHA on miRNA profiles in ALDH+BCs was analyzed by throughput sequencing. Upregulated and downregulated microRNAs are displayed in B) heatmaps and C) volcano plots. D,E) Expression of let‐7e‐5p and miR‐615‐3p in BC tissues versus adjacent normal tissues. J,K) The correlation of miR‐615‐3p expression and let‐7e‐5p expression with MICA expression and MICB expression were analyzed by bioinformatics. L,M) Luciferase assay of miR‐615‐3p and let‐7e‐5p. N,O) The effects of miR‐615‐3p and let‐7e‐5p on MICA and MICB were analyzed by flow cytometry. Data are presented as the mean ± SD, (*n*  =  3). *p < 0.05, **p < 0.01, and ***p < 0.001.

Next, we exposed ALDH1+ BCSCs to different concentrations of SAHA and found that SAHA increased MICA/B levels in a dose‐dependent manner (**Figure** **3**A). Mounting evidence has revealed that SAHA exerts anticancer effects by epigenetically regulating oncogenic miRNAs, which can regulate MICA/B expression in cancer cells.^[^ ^21^, ^36^ ^]^ Therefore, we hypothesized that SAHA may upregulate MICA/B expression in BCSCs via inhibition of MICA/B‐targeting miRNAs. To test this hypothesis, we first utilized high‐throughput sequencing to analyze the effect of SAHA on miRNA profiles in BCSCs. Hierarchical clustering showed that SAHA dramatically altered miRNA profiles in BCSCs, with 135 miRNAs downregulated (Figure 3B,C and Table S1, Supporting Information). Then, using the mirWalk and TargetScan databases, we predicted that 8 miRNAs, miR‐873‐3p, miR‐4454, let‐7e‐5p, miR‐30c‐1‐3p, miR‐615‐3p, miR‐92a‐3p, miR‐760 and let‐7c‐5p, have binding sites in MICA or MICB mRNA (Figure S2 and Table S2, Supporting Information). Among these miRNAs, let‐7e‐5p and miR‐615‐3p were highly expressed in BC tissues versus adjacent normal tissues (Figure 3D,E) and in TNBC cell lines, especially in ALDH1+ cells (Figure 3F,G). Moreover, high expression levels of let‐7e‐5p and miR‐615‐3p closely correlate with worse prognosis in BC patients (Figure 3H,I), implying the oncogenic potential effect of let‐7e‐5p and miR‐615‐3p in BC. Importantly, we also revealed an adverse correlation of miR‐615‐3p and let‐7e‐5p expression with MICA and MICB expression, respectively (Figure 3J,K). Thus, we wondered whether SAHA increases MICA/B expression in BCSCs by inhibiting miR‐615‐3p and let‐7e‐5p. It is well known that mature miRNAs are integrated into an RNA‐inducing silencing complex and bind to the 3’‐untranslated regions (3’‐UTRs) of specific target mRNAs to repress gene expression either by restraining translation or accelerating mRNA degradation. Based on this, to determine the regulatory potential of miR‐615‐3p on MICA expression, we first transfected BCSCs with luciferase reporter vectors containing the wild‐type 3’‐UTR of MICA or the 3’‐UTR with a mutated binding site of MICA in combination with the miR‐615‐3p mimic. As shown in Figure 3L, the miR‐615‐3p mimic markedly reduced luciferase activity of the wild‐type MICA construct compared to the control miRNA, but it did not affect luciferase activity of the MICA construct with a mutated binding site. For ascertaining the regulatory potential of let‐7e‐5p on MICB expression, luciferase reporter vectors containing the wild‐type 3’‐UTR of MICB or the 3’‐UTR with a mutated binding site of MICB coupled with the let‐7e‐5p mimic were transfected into BCSCs. It was observed that the let‐7e‐5p mimic markedly decreased luciferase activity of the wild‐type MICB construct compared to the control miRNA, whereas it had no influence on luciferase activity of the MICB construct with a mutated binding site (Figure 3M). These results verified that miR‐615‐3p and let‐7e‐5p directly bind to the MICA 3’‐UTR and MICB 3’‐UTR, respectively. Then, we utilized flow cytometry to analyze expression of MICA/B in BCSCs after transfection with miR‐615‐3p mimic or let‐7e‐5p mimic. As presented in Figure 3N–O, the miR‐615‐3p mimic significantly mitigated the SAHA‐mediated upregulation of MICA expression, and the let‐7e‐5p mimic alleviated SAHA‐mediated upregulation of MICB expression in BCSCs. Overall, these results confirm that SAHA upregulates MICA/B expression via inhibition of miR‐615‐3p and let‐7e‐5p in BCSCs. As expected, we further verified that SAHA profoundly augmented NK cell cytotoxicity to BCSCs (Figure S3, Supporting Information). Hence, there is a possibility that SAHA may enhance UCB‐NK cell infusion efficacy in preventing postresection relapse of CSC‐enriched TNBC.

### 2.3 Autophagy Inhibition Boosts SAHA‐Meditated Enhancement of NK Cell Cytotoxicity in BCSCs

**Figure 4:**
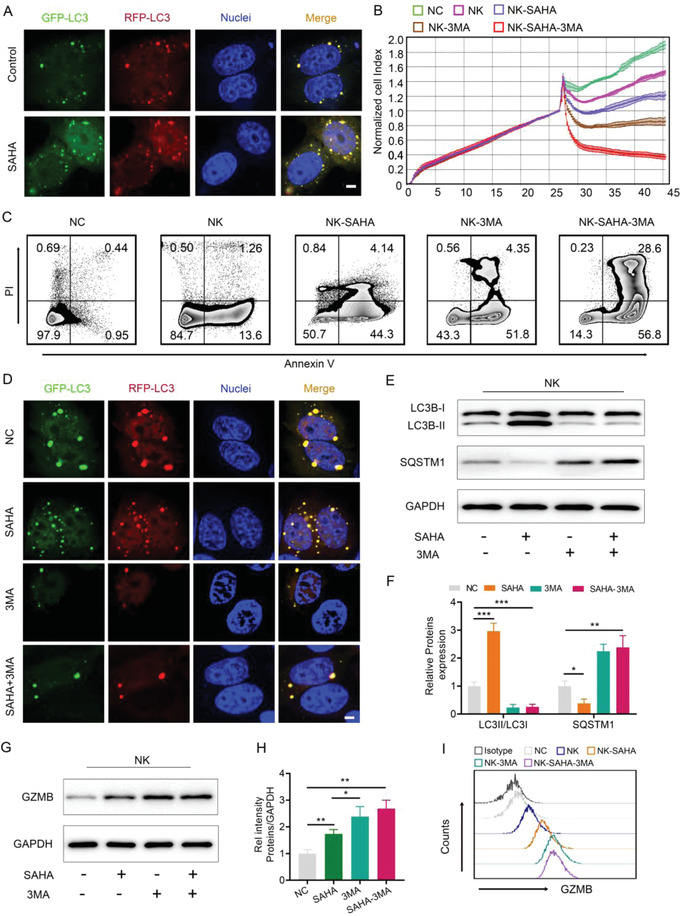
Autophagy inhibition augments the SAHA‐mediated enhancement of NK cell cytotoxicity to BCSCs. A) The autophagy level of ALDH^+^ cells modulated by SAHA, Scale bar = 2 µm. B) The cytotoxic effect of UCB‐NK cells on ALDH^+^ cells in the presence or absence of SAHA or 3MA. C) The level of apoptosis caused by UCB‐NK cells to ALDH^+^ cells in the presence or absence of SAHA or 3MA. D) The autophagy level of ALDH^+^ cells modulated by SAHA or 3MA, Scale bar = 2 µm. E,F) The levels of LC3B and SQSTM1 regulated by SAHA or 3MA were detected by western blot analysis. G,H)The levels of GZBM regulated by SAHA or 3MA were detected by western blot analysis. I) GZBM was analyzed by flow cytometry. Data are presented as the mean ± SD (*n*  =  3). *p < 0.05, **p < 0.01, and ***p < 0.001.

Autophagy is a lysosomal degradation process in cells that plays an essential role in degrading senescent organelles and aggregate‐prone proteins, as well as in clearing pathogens. Accumulated evidence has revealed that autophagy is dysregulated in multiple cancers and contributes to tumor development and progression.^[^ ^37^, ^38^, ^39^ ^]^ In particular, autophagy has been found to be elevated in BC cells, especially BCSCs, and serves as a pivotal driver of BC progression.^[^ ^4^ ^]^ Additionally, we observed higher basal autophagy levels in ALDH1+ BCSCs than in ALDH1‐ BC cells (Figure S4, Supporting Information). Notably, an inverse correlation between histone acetylation and autophagy in cancer stem cells (CSCs) has been reported.^[^ ^40^, ^41^ ^]^ Therefore, we explored whether SAHA, a histone deacetylase inhibitor, affects autophagy in BCSCs and observed that SAHA substantially upregulated autophagy levels in BCSCs (**Figure** **4**A and Figure S5A, Supporting Information). Interestingly, it has been reported that autophagy in cancer cells can reduce NK cell cytotoxicity, resulting in tumor immune escape.^[^ ^22^ ^]^ Hence, we hypothesized that SAHA‐induced autophagy may impede its maximum enhancement of NK cell cytotoxicity to BCSCs. To test this hypothesis, we first cocultured BCSCs with UCB‐NK cells in the presence or absence of SAHA and/or the autophagy inhibitor 3MA and then evaluated NK cell‐mediated killing of BCSCs using flow cytometry and the xCELLigence RTCA instrument system. As shown in Figure 4B,C and Figure S5B,C (Supporting Information), SAHA‐mediated enhancement of NK cell cytotoxicity to BCSCs was improved when 3MA was simultaneously added to the culture medium. Then, we further examined autophagic activity in BCSCs treated with SAHA and/or 3MA. Confocal microscopy identified fewer yellow puncta in BCSCs treated with both SAHA and 3MA than in cells only exposed to SAHA (Figure 4D and Figure S5D, Supporting Information), implying that there were fewer autophagosomes. LC3 and SQSTM1 have been most commonly used as markers of autophagosome formation,^[^ ^42^ ^]^ so we also detected their levels in BCSCs following SAHA and/or 3MA treatment. Western blot analysis showed that SAHA significantly increased LC3‐II and reduced SQSTM1 levels, but these effects were abrogated by 3MA (Figure 4E,F). These data indicate that 3MA efficiently represses SAHA‐induced autophagy in BCSCs. Given that autophagy in cancer cells inhibits NK cell cytotoxicity primarily by degrading NK cell‐derived granzyme B (GZMB),^[^ ^22^ ^]^ we further examined GZMB levels in BCSCs cocultured with NK cells in the presence or absence of SAHA and/or 3MA. As expected, GZMB levels were much higher in BCSCs treated with SAHA and 3MA than in those treated with SAHA alone (Figure 4G–I). In addition, we investigated the direct effect of SAHA and 3MA on NK cells. Figure S6A (Supporting Information) shows that NK cell viability was not affected when cells were cultured in medium supplemented with SAHA and 3MA. Meanwhile, NK cell‐derived GZMB, TNF‐*α* and IFN‐*γ* exhibited similar levels between the SAHA‐3MA treatment and the control groups (Figure S6B–D, Supporting Information). Thus, SAHA and 3MA did not affect NK cell function directly. Taken together, these results verified that 3MA substantially improves SAHA‐mediated enhancement of NK cell cytotoxicity via autophagy inhibition in BCSCs, indicating that UCB‐NK infusion with coadministration of SAHA and 3MA would be a more optimized protocol for treating TNBC.

### 2.4 Fabrication and Characterization of the Gel‐OS/SAHA@3MA@MBGN Hydrogel

**Figure 5:**
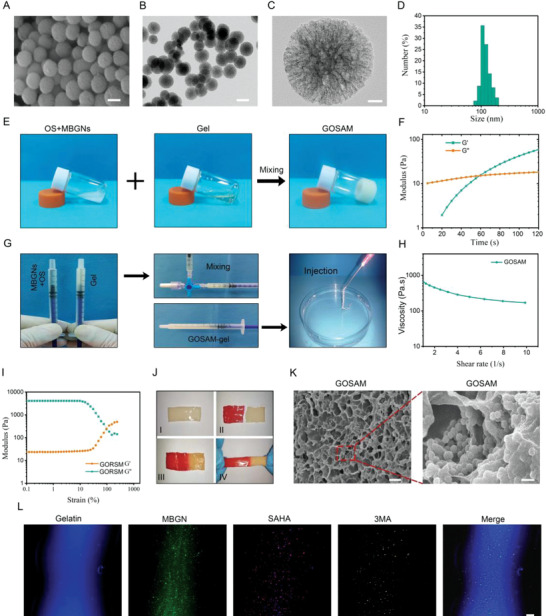
Characterization of GOSAM. A) Scanning electron microscope (SEM) images of mesoporous bioactive glass nanoparticles (MBGNs). Scale bar = 100 nm. B,C) Transmission electron microscopy (TEM) low‐ and high‐magnification images of MBGN. Scale bar in (B) = 100 nm; Scale bar in (C) = 20 nm. D) Average hydrodynamic size of MBGNs determined by Malvern Nano Zetasizer. E)Images show the mixing of gelatin (30% (w/v)) and OS (10% (w/v)) containing MBGNs (5% (w/v)) to form Gel‐OS/SAHA@3MA@MBGNs hydrogel (GOSAM) in a glass bottle at 37 °C. F) Dynamic time sweep rheological analysis to assess gelation kinetics of (GOSAM). The crossing point of storage modulus (*G*′) and loss modulus (*G*′′) is regarded as the gelation time. G) GOSAM can be easily injected into water via syringe and sustain a gel status. H) Shear‐thinning test of GOSAM. I) The strain amplitude sweep test (0.1–1000%) at a fixed angular frequency (1 rad s^−1^) at 37 °C. J) The self‐healing capacity of GOSAM. K) Scanning electron microscope (SEM) images of GOSAM. Left scale bar = 2 µm and right scale bar = 200 nm. L) Representative fluorescent images of GOSAM, in which gelatin was labeled with DAPI, MBGNs with FITC, SAHA with PI and 3MA with PerCP‐Cy5.5. Scale bar = 100 µm.

To fabricate the Gel‐OS/SAHA@3MA@MBGN hydrogel (GOSAM), oxidized starch (OS) was first generated as previously reported.^[^ ^43^ ^]^ In the Fourier transform infrared (FTIR) spectrum of starch, there was a new infrared band at about 1731 cm^−1^ (Figure S7, Supporting Information), which correlated with the presence of the aldehyde groups, verifying the successful oxidization of starch. Next, we synthesized biocompatible mesoporous bioactive glass nanoparticles (MBGNs) as described previously.^[^ ^33^ ^]^ Using scanning electron microscopy (SEM) and transmission electron microscopy (TEM), we observed that MBGNs displayed a porous microsphere structure with diameters of 100–200 nm (**Figure** **5**A–C). Additionally, Malvern Nano Zetasizer (UK) analysis confirmed that MBGNs exhibited diameters of 100–200 nm (Figure 5D). The X‐ray diffraction (XRD) pattern of MBGNs revealed a broad hump at approximately 2*θ* values of 20°–30°, revealing that MBGNs possess an amorphous structure (Figure S8, Supporting Information). MBGNs have been reported to be composed of 15 mol% CaO and 85 mol% SiO2.^[^ ^33^ ^]^ Consistent with this, element mapping analysis revealed the presence of Si, Ca, and O in MBGNs (Figure S9, Supporting Information). Additionally, we observed a sharp absorption peak at about 802 cm^−1^ and a broad peak between 1020 and 1242cm^−1^ in the FTIR spectrum of MBGNs (Figure S10, Supporting Information). These two peaks indicated the existence of Si—‐O‐Si and Si‐O chemical bonds, respectively.^[^ ^33^ ^]^ Then, SAHA and 3MA were introduced into MBGNs to obtain SAHA and 3MA‐loaded MBGNs (SAHA@3MA@MBGNs). The loading capacities of SAHA and 3MA are 59.02% and 51.2%, respectively (Figure S11, Supporting Information), which were calculated based on the drug concentrations of solutions measured before and after the loading processes by HPLC.

Additionally, the results of fluorescein isothiocyanate (FITC)‐labeled drug models showed that the loading capacities of SAHA and 3MA are 67.95% and 54.03%, respectively (Figure S12, Supporting Information), which was basically consistent with the results of HPLC. Finally, gel (30% (w/v)) and OS (10% (w/v)) with SAHA@3MA@MBGNs (5% (w/v)) were mixed at 37 °C to fabricate the GOSAM (Figure 5E). We monitored the gelling process of the hydrogel through the dynamic time sweep rheological experiment and found that a crossover of loss modulus (*G*′′) and storage modulus (*G*′) occurred 60 s after mixing, indicating the gelation of GOSAM (Figure 5F). There are at least two chemical mechanisms for the gelation of GOSAM. On the one hand, the Schiff base bond efficiently contributes to chemical crosslinking in the polymer hydrogels.^[^ ^44^ ^]^ Strikingly, the FTIR spectrum of GOSAM revealed that the Schiff base bond formed between the aldehyde groups in OS and the amino groups in gelatin (Figure S13, Supporting Information). On the other hand, an abundance of silanol groups (Si‐OH) form in MBGNs due to the hydration reaction when MBGNs are immersed in an aqueous environment, and then silanol groups can interact with the amino groups in gelatin to consolidate the crosslinking in the polymer hydrogel.^[^ ^33^, ^34^ ^]^

Injectability is a prerequisite of hydrogel‐based cancer therapy in terms of clinical manipulation, so the injectability of GOSAM was further evaluated. As illustrated in Figure 5G, the GOSAM precursor could be glibly injected into water using a tiny syringe and formed a steady gel in situ, implying the excellent injectability of GOSAM. In particular, the viscosity of GOSAM measured via the rheometer revealed a gradual decrease with increasing shear rate (Figure 5H), implying that the injectability of the hydrogel may be at least partly attributed to the shear‐induced destruction of the dynamic hydrogel network. In addition to the injectability, the self‐healing capacity should also be seriously considered because it closely correlates with the lifetime and function of hydrogels applied to the defect tissues or surgical bed.^[^ ^45^ ^]^ Hence, we further evaluated the self‐healing properties of GOSAM. For this purpose, the strain amplitude sweep experiment was first performed at 37 °C, in which the strain varied from 0.1% to 1000%. As shown in Figure 5I, the *G*′ and *G*″ values of GOSAM remained stable as the strain gradually increased when the strain was below 10%. Nevertheless, a marked drop in the values of *G*′ and *G*″ occurred as the strain further increased, and they became equal at approximately 100% strain. These results demonstrate that GOSAM experienced structural destruction once the critical strain of its network was surpassed, indicating its potential self‐healing capacity. Then, we performed a macroscopic self‐healing test to validate the self‐healing efficiency of GOSAM. Briefly, a linear GOSAM was first cut into two sections, which were placed together at 37 °C for 5 min with no stimuli. As shown in Figure 5J, the two sections healed as a whole gel and remained intact under the pull force. The existence of the dynamic reversible Schiff's base bond network between the aldehyde groups in oxidized starch and the amine groups in gelatin may mostly explain the excellent self‐healing capacity of GOSAM. SEM was utilized to observe the structure of GOSAM. Representative images of the hydrogel revealed that GOSAM displays a porous structure with a rough pore surface (Figure 5K). To analyze the spatial distribution of SAHA and 3MA in the hydrogel, we first used DAPI‐labeled gelatin, FITC‐labeled MBGNs, PI‐labeled SAHA, and PerCP‐Cy5.5‐labeled 3MA to synthesize GOSAM. Then, sections of GOSAM were created and subjected to confocal scanning analysis. As shown in Figure 5L, the FITC signal from MBGNs displays a uniform distribution pattern in GOSAM and is colocalized with SAHA and 3MA‐derived signals. These data confirm that we successfully fabricated GOSAM.

### 2.5 Biocompatibility and Biodegradation of Gel‐OS/SAHA@3MA@MBGN Hydrogel

**Figure 6:**
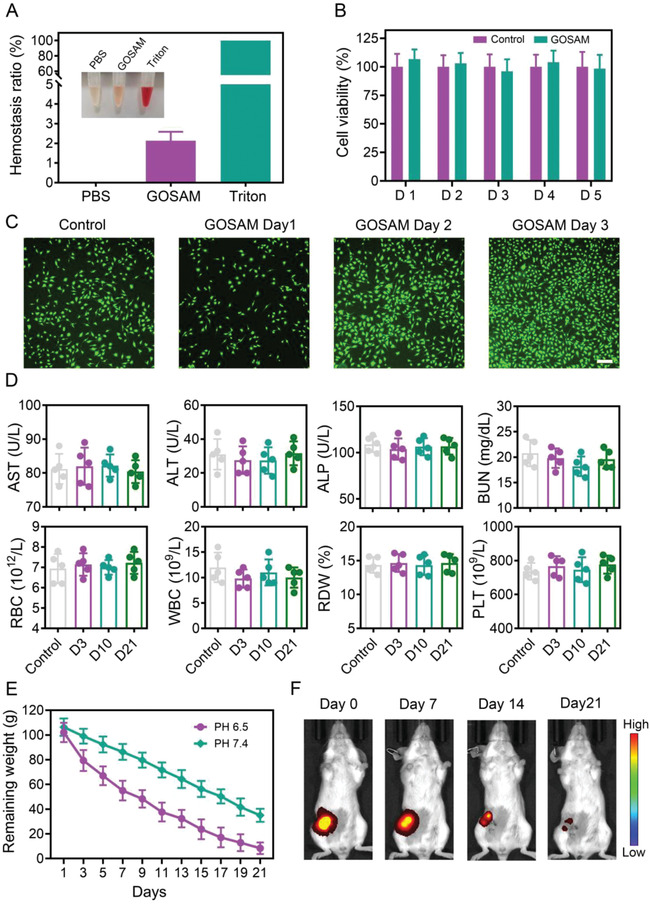
Biocompatibility and biodegradation of GOSAM. A) The hemocompatibility assay for GOSAM, *n* = 3. B) Cell viability of MCF‐10A cells cocultured with GOSAM. C) Live/dead staining assay of MCF‐10A cells cocultured with GOSAM, scale bar = 100 µm. D) Serum levels of biomarkers of liver function and kidney function and blood cell parameters after the application of GOSAM into mouse breast pads, *n* = 5. E) The degradation of GOSAM during incubation in pH 6.5 and pH 7.4 PBS, *n* = 3. F) Fluorescence IVIS imaging monitoring the in vivo degradation of Cy5.5‐labeled GOSAM after injected into mouse breast pads. Data are presented as the mean ± SD (*n*  =  3). *p < 0.05, **p < 0.01, and ***p < 0.001.

The biocompatibility of hydrogels is a considerable issue in terms of their suitability as drug delivery carriers. First, hemolysis experiments were conducted to assess the hemocompatibility of GOSAM, in which Triton X‐100 and phosphate buffer saline (PBS) solution were used as the positive control and the control groups, respectively. As shown in **Figure** **6**A, the solution turned red in the Triton X‐100 group but remained bright in the GOSAM and the PBS groups. Of note, the hemolysis ratio of the GOSAM group was below 5%, which is widely considered the criterion for good hemocompatibility.^[^ ^32^ ^]^ Next, we plated human normal mammary epithelial cells (MCF‐10A) into 96‐well plates coated with or without hydrogel to perform cell viability and live/dead staining assays. As shown in Figure 6B, GOSAM had no significant effects on MCF‐10A cell viability. The live/dead staining experiments revealed that there were almost no red cells after 1 to 3 d following incubation, whereas cells stained with green were extensively scattered across the entire field of vision in the GOSAM group (Figure 6C). These results implied the excellent cytocompatibility of the hydrogel. To investigate its biocompatibility in vivo, we subcutaneously injected GOSAM into mice and collected blood samples from these mice at the indicated time points for biochemical analysis. As shown in Figure 6D, GOSAM did not affect serum levels of hepatic function markers, including alanine aminotransferase (ALT), aspartate aminotransferase (AST) and alkaline phosphatase (ALP), or kidney function markers, such as blood urea nitrogen (BUN). In addition, GOSAM also had no influence on white blood cell (WBC) count, red blood cell (RBC) count, red cell distribution width (RDW) or platelet (PLT) count. Hence, these results revealed that GOSAM exhibits excellent biocompatibility in vivo.

To evaluate the biodegradation of GOSAM, we first immersed it in PBS (pH = 7.4 or 6.5) at 37 °C and weighed the remaining hydrogel at the indicated time points. The results showed that the weights of the residual GOSAM gradually declined at pH 7.4 or pH 6.5 (Figure 6E). Nevertheless, the degradation rate of the pH 6.5 group occurred more rapidly than that of the pH 7.4 group, indicating that GOSAM exhibits pH‐responsive degradation behavior. There are at least two potential chemical mechanisms for the pH‐sensitive degradation of hydrogels. First, the Schiff base bonds, as a type of dynamic chemical bond, can be broken into aldehyde groups and amino groups when they are challenged with acidic conditions.^[^ ^46^ ^]^ In addition, MGBNs are apt to break down due to their reaction with H^+^.^[^ ^33^ ^]^ Next, we subcutaneously injected GOSAM into mice and applied an in vivo bioluminescence imaging system to monitor its biodegradation. The results showed that the fluorescence signals of GOSAM gradually declined and were almost undetectable 21 d after injection (Figure 6F and Figure S14, Supporting Information). Additionally, we also made the general observation on the hydrogels subcutaneously injected into mice at day 1, day 7, day 14, and day 21. As shown in Figure S15 (Supporting Information), the hydrogel bulk gradually decreased with a subtle amount of residual on day 21, which was basically consistent with the results of the in vivo bioluminescence imaging experiments. In general, these results confirm that GOSAM exhibits acceptable degradation behavior in vivo.

### 2.6 Drug Release by the Gel‐OS/SAHA@3MA@MBGN Hydrogel

To investigate the drug release behavior, we first immersed GOSAM in pH 7.4 PBS solution or pH 6.5 PBS solution, which simulates physiological and acidic tumor microenvironment conditions, respectively. The drug concentrations of solutions at the indicated time points were measured by HPLC, based on which the drug release curves were established. The results showed that GOSAM allowed for the sustained release of SAHA and 3MA (Figure S16, Supporting Information). Notably, more rapid release of SAHA or 3MA was observed in pH 6.5 PBS solution than in pH 7.4 PBS solution. The cumulative release levels of SAHA were 29.6% in pH 7.4 PBS and 91.6% in pH 6.5 PBS solution 21 d after being immersed. The cumulative release levels of 3MA were 28.1% in pH 7.4 PBS solution and 92.3% in pH 6.5 PBS solution. In addition, the results of FITC‐labeled drug model further confirmed the pH‐responsive sustained drug release behavior of GOSAM (Figure S17A,B, Supporting Information). The pH‐responsive drug release behavior of GOSAM in vitro may be attributed to the pH‐sensitive rupture of the Schiff base bonds^[^ ^46^ ^]^ and the pH‐sensitive degradation of MGBNs.^[^ ^33^ ^]^ To evaluate the in vivo drug release, we first encapsulated Cy5.5‐labeled SAHA or 3MA into the hydrogels, and then injected the hydrogels into the tumor resection bed of the mouse model. Next, the in vivo imaging system was adopted to monitor the signals of Cy5.5‐labeled SAHA or 3MA at the tumor resection bed. As shown in Figure S17C (Supporting Information), their signals gradually faded and were sustained for at least 14 d. Overall, these data verify that GOSAM allows for a pH‐sensitive sustained release of SAHA and 3MA.

### 2.7 Gel‐OS/SAHA@3MA@MBGN Hydrogel Enhances UCB‐NK Cell Infusion Efficacy in a TNBC Mouse Model

**Figure 7:**
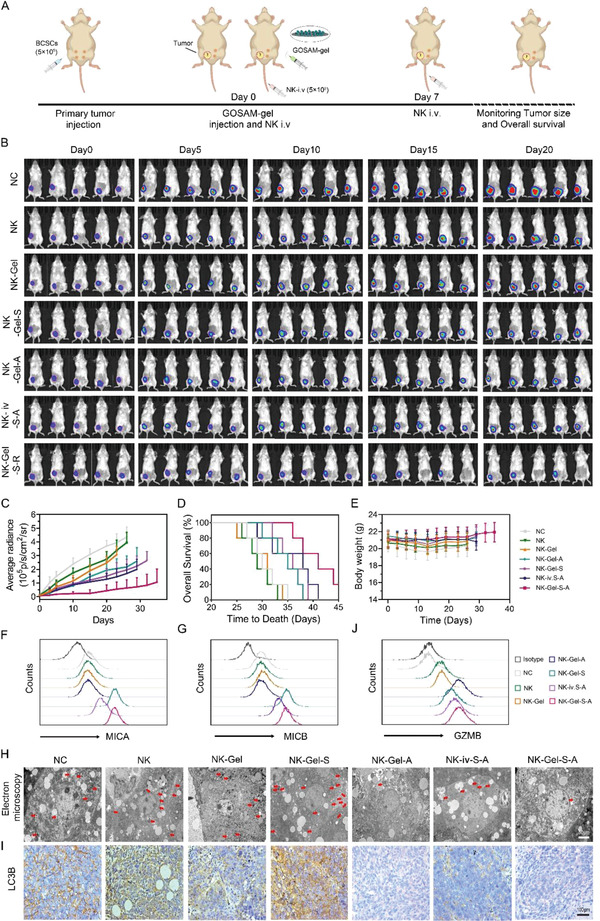
Prevention of BCSC progression by GOSAM. A) Schematic presenting the combined therapy of GOSAM and UCB‐NK‐cell infusion in the BCSC mouse model. B) Representative bioluminescence images of tumors after different treatments. C) Average tumor growth kinetics in different groups, stopping when the first mouse in each group died, for each group, *n* = 5. D) The overall survival rate of mice treated with different methods. Data were analyzed using the log‐rank test. E) Weight changes of mice treated with different methods, stopping when the first mouse in each group died, for each group, *n* = 5. F) MICA, G) MICB and J) GZMB expression in different groups analyzed by flow cytometry. H) The autophagosomes in different groups were analyzed using electron microscopy, scale bar = 50 µm. I) The autophagy level measured by immunohistochemical staining of LC3B, scale bar = 100 µm. Data are shown as the mean ± SD.

To evaluate the synergistic antitumor efficiency of UCB‐NK cell infusion and GOSAM, TNBC mouse models were established by inoculating ALDH1+ BCSCs into the breast pad. Then, these tumor‐bearing mice were randomly allocated into seven groups on day 0 (**Figure** **7**A), including the PBS (NC group), NK cell infusion alone (NK group), NK‐Gel (NK cell infusion with intratumor drug‐unloaded hydrogel injection), NK‐Gel‐S group (NK cell infusion with SAHA‐loaded hydrogel intratumor injection), NK‐Gel‐A group (NK cell infusion with 3MA‐loaded hydrogel intratumor injection), NK‐iv‐S‐A group (NK cell infusion with intravenous injection of SAHA and 3MA) and NK‐Gel‐S‐A (NK cell infusion with SAHA‐3MA‐loaded hydrogel intratumor injection). The dose of SAHA or 3MA in each group was equal. Before and after treatment, the tumor growth of each group was monitored using the in vivo bioluminescence imaging system. As shown in Figure 7B,C, tumor growth was slightly delayed in the NK and NK‐Gel groups, moderately inhibited in the NK‐Gel‐S, NK‐Gel‐A, and NK‐iv‐S‐A groups, and profoundly repressed in the NK‐Gel‐S‐A group compared to the PBS group. Kaplan‐Meier survival analysis with the log‐rank test was conducted to determine the overall survival (OS) rate of mice in each group. The Kaplan‐Meier curve showed that the OS rate of the NK‐Gel‐S‐A group was markedly higher than that of the other groups (Figure 7D). In addition, we observed that the mice in the NK‐Gel‐S‐A group exhibited no significant weight loss (Figure 7E), indicating no additional systemic toxicity from GOSAM treatment. Taken together, these results verified that GOSAM intratumor injection significantly improves UCB‐NK cell infusion efficacy to inhibit TNBC growth.

As mentioned above, GOSAM allowed for the sustained release of SAHA and 3MA, which increases MICA/B expression and inhibits autophagy in BCSCs. Thus, we explored MICA/B expression and autophagy levels in a TNBC mouse model after GOSAM treatment. Tumor tissues were collected from mice in each group and then subjected to flow cytometry analysis to evaluate MICA/B expression. The results showed that there was no significant upregulation of MICA/B expression in the NK, NK‐Gel, or NK‐Gel‐A groups with a slight increase in the NK‐iv‐S‐A group and a substantial elevation in the NK‐Gel‐S and NK‐Gel‐S‐A groups compared to the PBS group (Figure 7F,G). The autophagy levels in tumor tissues were examined by electron microscopy and immunohistochemical staining. As presented in Figure 7H,I and Figure S18 (Supporting Information), there were fewer autophagic vacuoles and reduced LC3B expression in the tumor tissues of the NK‐Gel‐A and NK‐Gel‐S‐A groups than in the tumor tissues from the other groups. These results indicate that GOSAM treatment is far superior to systemic administration of SAHA and 3MA in upregulating MICA/B expression and inhibiting autophagy in vivo. The advantage of GOSAM may be attributed to the fact that SAHA and 3MA encapsulated in the hydrogel exhibit elevated bioavailability. There is evidence that autophagy in tumor cells can resist NK cell‐mediated killing by degrading NK cell‐derived GZMB.^[^ ^22^ ^]^ Hence, we further isolated tumor cells from the resected tumor tissues and analyzed the GZMB levels in these cells using flow cytometry. In response to the results regarding autophagy, the GZMB levels in the NK‐Gel‐A and NK‐Gel‐S‐A groups were significantly higher than those in the other groups (Figure 7J). Collectively, these results confirmed that GOSAM efficiently upregulated MICA/B expression and inhibited autophagy in vivo. Therefore, enhancing NK cell infusion antitumor efficacy using GOSAM may be at least partly attributed to hydrogel‐mediated MICA/B upregulation and autophagy inhibition.

**Figure 8:**
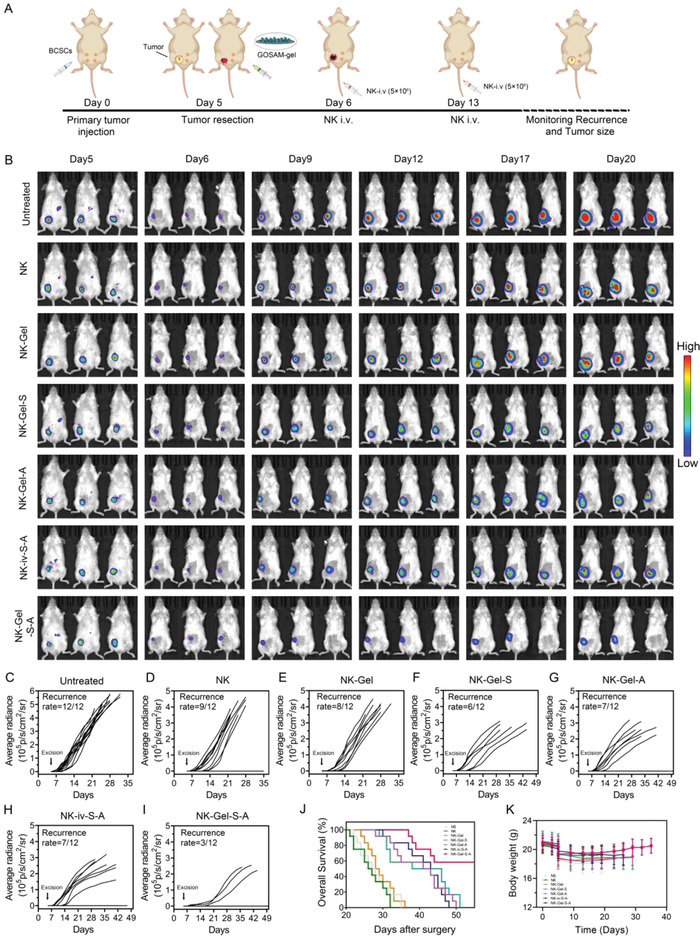
Prevention of tumor recurrence by GOSAM. A) Schematic illustrating the combined therapy of GOSAM and UCB‐NK cell infusion in a resection model of murine orthotopic TNBC. B) Bioluminescence images of recurrent tumors after operation resection in different groups. C–I) Individual tumor growth kinetics of mice in different groups. J) The overall survival rate of mice in different groups, data were analyzed with log‐rank test. K) Weight changes of mice treated with different methods, stopping when the first mouse in each group died, for each group, *n* = 12. Data are shown as the mean ± SD.

TNBC has a high risk of postresection relapse, which is primarily attributed to the presence of BCSCs in the residual foci.^[^ ^4^ ^]^ Hence, we further investigated whether in situ administration of GOSAM could improve UCB‐NK cell infusion efficacy in combating postsurgical relapse of TNBC (**Figure** **8**A and Figure S19, Supporting Information). To this end, TNBC mouse models were established by inoculating BCSCs into the breast pads of mice on day 0. Then, these mice were subjected to surgery to remove tumors on day 5 and allocated into seven groups in a random manner, including the untreated group (without additional treatments), NK (NK cell infusion alone), NK‐Gel (NK cell infusion with drug‐unloaded hydrogel injection onto the tumor resection bed), NK‐Gel‐S (NK cell infusion with SAHA‐loaded hydrogel injection), NK‐Gel‐A (NK cell infusion with 3MA‐loaded hydrogel injection), NK‐iv‐S‐A (NK cell infusion with the intravenous injection of SAHA and 3MA) and NK‐Gel‐S‐A (NK cell infusion with GOSAM injection). In vivo bioluminescence imaging was utilized to monitor tumor relapse in each group (Figure 8B). The results showed that mice in the untreated group had a relapse rate of 100% (12/12) within a 50 day observation period (Figure 8C), consistent with the published literature.^[^ ^47^ ^]^ The relapse rate was slightly reduced in the NK and NK‐Gel groups (Figure 8D,E), moderately decreased to approximately 50% in the NK‐S, NK‐A and NK‐iv‐S‐A groups (Figure 8F–H), and dramatically decreased to 25% (Figure 8I) in the NK‐Gel‐S‐A groups. Moreover, Kaplan‐Meier survival analysis with the log‐rank test showed that UCB‐NK infusion combined with GOSAM injection improved the overall survival of mice most significantly among all the therapeutic modalities (Figure 8J). In addition, a weight increase in the mice of the NK‐Gel‐S‐A group was observed (Figure 8k), suggesting that GOSAM treatment caused no additional systemic toxicity. Therefore, these results demonstrated that in situ injection of GOSAM efficiently enhanced UCB‐NK cell infusion to combat postresection relapse of TNBC.

### 2.8 Hemostatic Performance of the Gel‐OS/SAHA@3MA@MBGN Hydrogels

**Figure 9:**
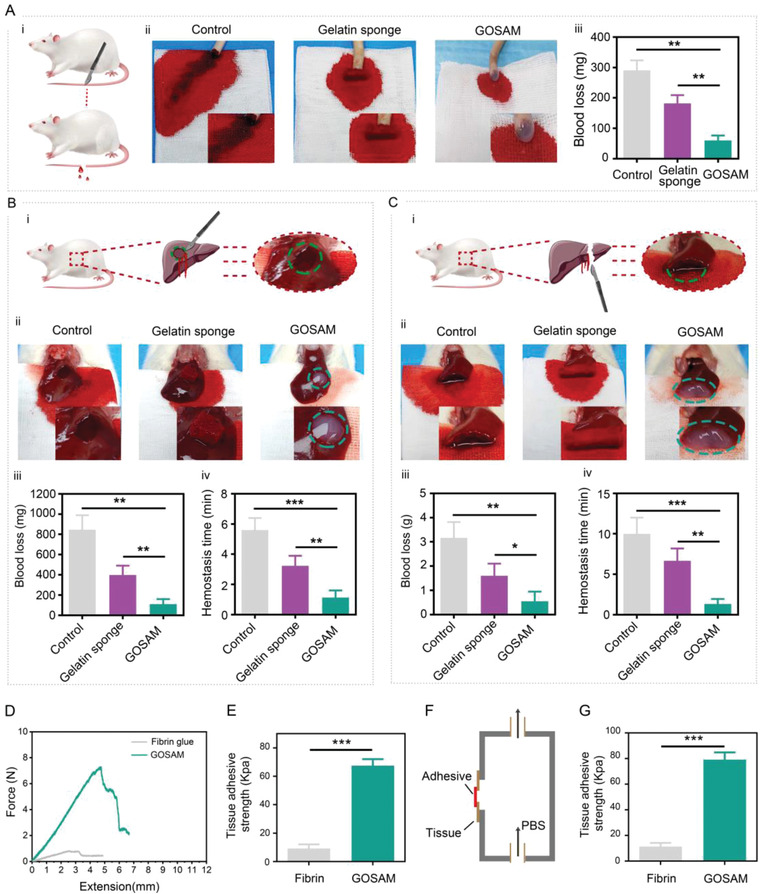
Hemostatic property and adhesive capability of GOSAM. A) Rat‐tail amputation bleeding model, *n* = 3. B) rat liver slice resection wound model and C) rat partial hepatectomy wound bleeding models: I) Schematic diagram of different models; II) Representative photos and quantitative statistics of III) blood loss and IV) hemostasis time for different groups, *n* = 3. D) Representative lap‐shear adhesion curves of GOSAM and commercially available fibrin glue applied to porcine skin. E) Adhesive strength of GOSAM compared with that of fibrin glue. F) Schematic diagram of the assay for burst pressure measurements. G) Burst pressure of GOSAM to liver, *n* = 3. Data are presented as the mean ± SD. *p < 0.05, **p < 0.01, and ***p < 0.001.

There is growing evidence that intraoperative bleeding during tumor resection facilitates the invasion of malignant cells into blood circulation, consequently increasing the risk of local tumor relapse.^[^ ^48^ ^]^ Efficient and timely hemostasis during surgery would thus make an additional contribution to preventing tumor relapse. In clinical practice, conventional physical compression and thermal hemostasis are routinely applied to control intraoperative bleeding. However, these methods perform poorly in reducing blood loss from regions that are difficult to access. Notably, topical hemostatic implants may show promise for controlling intraoperative bleeding in this scenario, such as injectable polymer hydrogels with anticancer agents.^[^ ^49^ ^]^ Hence, we further explored the hemostatic performance of GOSAM. To this end, a hemostasis experiment was first conducted in a mouse tail amputation bleeding model (**Figure** **9**A‐i). The representative images in Figure 9A‐ii show reduced blood loss in the GOSAM group than in the control and commercial gelatin sponge groups. Consistently, the quantitative results showed that the blood loss of the GOSAM group was lower than that of the other two groups (Figure 9A‐iii). Given that the liver is an organ with a rich supply of blood, we also utilized liver bleeding models to further evaluate the hemostatic effect of GOSAM. A rat liver slice resection wound model was used (Figure 9B‐i). The representative images in Figure 9B‐ii show that the gauze was significantly stained with blood in the control group, moderately stained with blood in the commercial gelatin sponge group, and only slightly stained with blood in the GOSAM group. The quantitative results revealed massive blood loss in the control group, median hemorrhage in the gelatin sponge group, and minimal bleeding in the GOSAM group (Figure 9B‐iii). In addition to reducing the amount of blood loss, it is also of great importance to quickly control bleeding during surgery. Hence, we also determined the hemostasis time of each group. As expected, GOSAM substantially shortened the time to control bleeding (Figure 9B‐iv). In addition to the rat liver slice resection wound model, a bleeding model of partial hepatectomy in rats was also used to evaluate the GOSAM‐mediated hemostasis effects (Figure 9C‐i). The results showed that GOSAM treatment yielded faster and better hemostasis versus the control and commercial gelatin sponge treatments in this model (Figure 9C‐ii–iv). These results demonstrate that GOSAM has good hemostasis function, indicating that it could also be used as an intraoperative hemostat, in addition to an antitumor drug delivery platform.

The aldehyde groups in hydrogels can covalently react with the amino groups on the surfaces of freshly injured tissues to generate tight bonding at the interface, which mediates powerful adhesion of the hydrogels to the injured fresh tissues. Of note, the wet tissue adhesion of hydrogels triggers the formation of physical barriers to seal the bleeding sites, leading to rapid hemostasis.^[^ ^45^ ^]^ Strikingly, aldehyde groups exist in oxidized starch, one of the synthesized materials of GOSAM. Hence, we examined whether wet tissue adhesion could be one of the mechanisms for GOSAM‐mediated hemostasis. To address this issue, we first assessed the tissue adhesive capacity of GOSAM using the lap‐shear adhesion test and compared it to that of commercial fibrin glue. As presented in Figure 9D,E, the adhesive strength of GOSAM (67.5 ± 3.7 kPa) was significantly greater than that of the fibrin glue (9.26 ± 2.7 kPa) and that of the adhesive hemostatic hydrogel we previously developed.^[^ ^45^ ^]^ In addition, the burst pressure test was conducted to evaluate the adhesion of GOSAM. Figure 9F,G shows that GOSAM had a much more powerful burst pressure than fibrin glue. Overall, these results demonstrate that GOSAM has an excellent wet tissue adhesive capacity, which may partly explain its hemostatic effect.

### 2.9 Wound Healing Function of Gel‐OS/SAHA@3MA@MBGN Hydrogels

**Figure 10:**
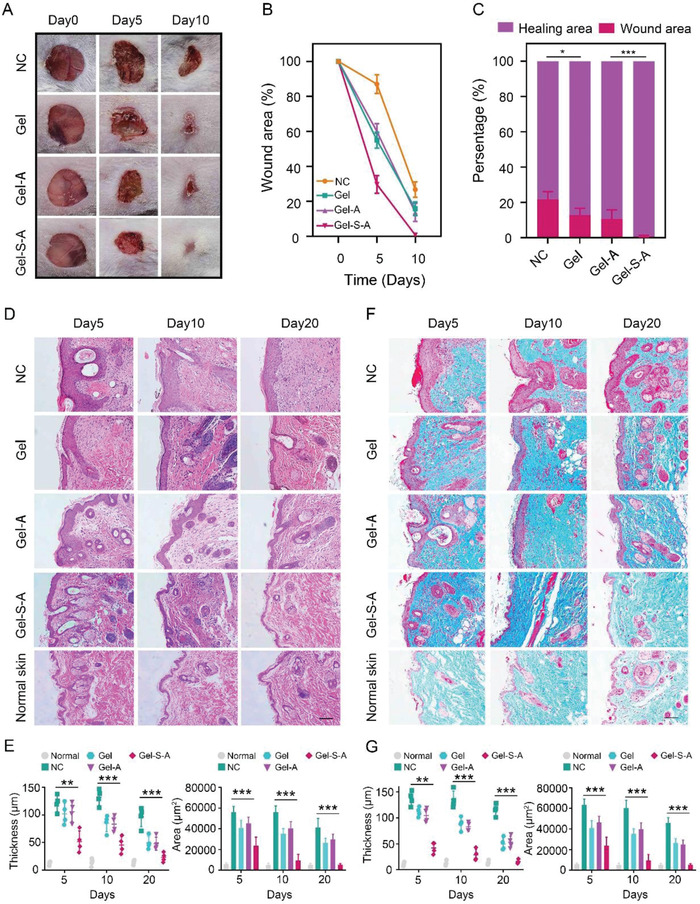
Representative images of wound healing in mice treated with different methods. A) Representative images of wound healing of mice treated with different methods, *n* = 3. B) Quantification of wound defects on Days 0, 5, and 10, *n* = 3. C) The proportions of wound defect and residual area after 20 d, *n* = 3. D) HE staining images of normal skin and wound area in diverse groups, scale bar = 100 µm. E) Quantification of the epidermal thickness and area of diverse groups from the HE staining, *n* = 4. F) Masson staining images of normal skin and wound area in diverse groups, scale bar = 100 µm. G) Quantification of the epidermal thickness and area of diverse groups from Masson staining, *n* = 4. Data are presented as the mean ± SD. *p < 0.05, **p < 0.01, and ***p < 0.001.

Curative treatment is the ultimate goal for BC patients, but postoperative wound healing is also a non‐negligible issue since its impairment increases the risk of postoperative complications and even causes detrimental psychological effects.^[^ ^50^ ^]^ There is evidence that porous bioactive glass nanoparticles promote the migratory capacity of epidermal cells by releasing Ca ions and accelerating wound healing.^[^ ^51^ ^]^ In addition, SAHA has been reported to facilitate wound healing in a mouse model.^[^ ^50^ ^]^ Hence, we further explored the effect of GOSAM on wound healing. Full‐thickness skin defect mouse models were first established, and mice were subsequently randomly divided into four groups, including NC (no treatments), Gel (drug‐unloaded hydrogel injection into wound defects), Gel‐A (3MA‐loaded hydrogel injection) and Gel‐S‐A (GOSAM injection) groups. The representative images showed that the wound defect was almost healed 10 d after GOSAM treatment (**Figure** **10**A). On day 5, the remaining wound area of the NC group was 87.4%, while those of the Gel, Gel‐A and Gel‐S‐A groups were 54.8%, 59.5%, and 29.6%, respectively. Thus, all Gel groups exhibited the ability to promote wound healing, with Gel‐S showing the strongest effect (Figure 10B,C). From these results, we observed better wound healing of skin in the Gel group than in the NC group, which may be attributed to the promotive effect of MBGNs on epidermal cells. Additionally, skin repair in the Gel‐S‐A group was superior to that in the Gel‐A group, indicating that SAHA promotes wound healing.

Then, H&E staining experiments were conducted to quantitatively evaluate the epidermal thickness and area of the different groups to further evaluate the degree of healing in the skin. The results showed that the Gel‐S‐A group had the lowest epidermal thickness and area after 20 d of treatment (Figure 10D,E). In addition, skin tissue in the Gel‐S‐A group displayed the most similar tissue structure to normal skin tissue. It is well known that an increase in collagen contributes to wound healing during the early stage, while excessive and persistent collagen deposition causes scar tissue formation, affecting skin repair.^[^ ^52^ ^]^ Thus, we also performed Masson staining experiments to assess collagen deposition and the healing level of skin in each group. As illustrated in Figure 10F, the collagen deposition in the Gel‐S‐A group dramatically increased during the early stage but declined in the late stage, and this trend was the most prominent in the Gel‐S‐A group among all the groups. Additionally, the epidermal thickness and area were the lowest 20 d after GOSAM injection (Figure 10G), and the regenerated skin tissue in the Gel‐S‐A group mostly resembled normal tissue structure (Figure 10F). Taken together, these results demonstrate that GOSAM injection allows for efficient skin repair, indicating its potential for accelerating postoperative wound healing in BC patients.

## 3 Experimental Section

### Materials and Reagents

Ammonia solution, starch, gelatin, and sodium periodate were obtained from Aladdin (Shanghai, China). Hexadecyl trimethyl ammonium bromide, calcium nitrate, ethyl acetate, absolute ethanol, suberoylanilide hydroxamic acid (SAHA), and 3‐methyl adenine (3MA) were acquired from Sinopharm Chemical Reagent Co., Ltd. (Shanghai, China), Tianjin Fuchen Chemical Reagent Factory, Guangdong Guanghua Technology Co., Ltd., Sigma–Aldrich (Saint Louis, US) and GLPBio (Montclair, US), respectively. DAPI, PI, Cy5.5, and FITC were purchased from KeyGEN BioTECH (Nanjing, China).

### Cell Lines and Animals

Human TNBC cell lines were obtained from ATCC. Female NOD‐SCID IL‐2 receptor gamma null (NSG) mice (6–8 weeks, 18–22 g) and SD rats (male, 250–300 g) were purchased from GemPharmtech Biotechnology Co. Ltd. and Laboratory Animal Center of Southern Medical University, respectively. The animal experiments were implemented with reference to the protocols approved by the Animal Care and Use Committee of Sun Yat‐Sen University (No. IACUC‐ F3‐21‐0901).

### Quantitative Reverse Transcription PCR (qRT‐PCR) and Western Blotting

Quantitative reverse transcription‐PCR (qRT‐PCR) and western blotting were performed as previously reported.^[^ ^19^, ^42^ ^]^ MiRNA expression was normalized to RNU6B according to the 2^−ΔΔct^ method. Quantitative PCR was implemented utilizing Universal SYBR qPCR Master Mix (Vazyme, Q511‐02). The detailed staining protocol was previously described. Anti‐LC3B, anti‐GZMB and anti‐SQSTM1 were purchased from Cell Signaling Technology (Danvers, US).

### Immunohistochemistry

Immunohistochemistry was performed as previously described.^[^ ^53^ ^]^ In brief, antibodies against LC3B were tested on sections from the excised xenograft breast cancer tissues. Then, IHC staining of paraffin‐embedded tissues with antibodies against LC3B (1:100; ABclonal) was scored to evaluate LC3B expression levels.

### Immunofluorescence and Confocal Microscopy

To monitor autophagic flux, MDA‐MB‐231 cells treated with SAHA and/or 3MA were transfected with mRFP‐GFP‐tagged LC3 for 24 h using Lipofectamine 3000 (Thermo Fisher). Images were obtained using a confocal microscope (ZEISS, Germany) with a 63× oil objective. The number of yellow dots (RFP+GFP+) per cell was analyzed to evaluate autophagic activity. To determine the stemness of ALDH^+^ MDA‐MB‐231 cells and ALDH^−^ MDA‐MB‐231 cells, these cells were first attached to coverslips coated with poly‐l‐lysine and then fixed them in 4% paraformaldehyde. After washing three times using PBS solution, MDA‐MB‐231 cells were first incubated with 1% fetal bovine serum and subsequently with primary antibodies against Sox‐2 (1:200, Cell Signaling Technology, US), *α*‐SMA (1:200, Abcam, UK), Nanog (1:200, Cell Signaling Technology, US), Oct‐4 (1:200, Cell Signaling Technology, MA) and CK‐18 (1:200, GeneTex, US), as well as a secondary antibody (1:500, Beyotime, China). In addition, the nuclei using DAPI (Invitrogen, CA) was counterstained. Immunofluorescence images were acquired using a laser confocal scanning microscope (ZEISS, Germany).

### Flow Cytometry

For analysis of surface MICA and MICB expression, cells (5 × 10^5^) were incubated with anti‐human MICA phycoerythrin mAb (SinoBio, Shanghai, China) and anti‐human MICB phycoerythrin mAb (SinoBio, Shanghai, China) at 4 °C for 30 min. For the intracellular staining of GZMB, CELL STIM+TRANS INHIB CKL (eBioscience, US) was added for stimulation for 4 h and incubated with anti‐GZMB antibody (eBioscience, US) for 30 min. For analysis of TNF‐*α* and IFN‐*γ* on NK cells, CD3^−^CD56^+^ (eBioscience, US) cells were first isolated and incubated them with TNF‐*α* and IFN‐*γ* (eBioscience, US) for 30 min. The cells were then washed three times with PBS. Fluorescence was detected using fluorescence‐activated cell sorting (FACS) and analyzed using FlowJo software. The Annexin V/PI staining protocol was previously described.^[^ ^54^ ^]^

### DNA Constructs and Luciferase Assay

Luciferase reporters were established by inserting the wild‐type or mutated 3′‐UTR of MICA and MICB at the 3′ end of the open reading frame region in the pGL3 luciferase reporter vector. To conduct luciferase assays, ALDH1+ MDA‐MB‐231 cells were first planted in a 96‐well plate with 1 × 10^4^ cells in each well. After 8 h of cell attachment, these cells were cotransfected with 50 ng of MICA or MICB reporter constructs coupled with either 50 × 10^−9^ m of let‐7e‐5p or miR‐615‐3p mimics or their control miRNAs. Then, the Reporter Assay System Kit was applied to measure the luciferase activity in cells after 48 h of incubation. The detailed procedures of this assay were described previously.^[^ ^19^ ^]^

### MiRNA High‐Throughput Sequencing and Sequencing Data Analysis

The miRNA high‐throughput sequencing and data analysis services were provided by LC Sciences (Hangzhou, China). Total RNA was extracted from ALDH1+ MDA‐MB‐231 cells treated with SAHA or untreated cells. TruSeq sRNA Sample Preparation Kits (Illumina, USA) were applied to construct miRNA‐sequencing libraries from 1 µg of extracted total RNA per sample based on the manufacturer's instructions. Then, miRNA‐sequencing libraries were barcoded and merged together for miR‐seq on an Illumina HiSeq‐2000/2500 platform, which allows for 3–5 million 50 bp single‐end reads. A quality control program was performed to obtain clean reads from raw sequencing data as previously described, which were subsequently mapped against the Rfam database and Repbase database to annotate miRNAs. Finally, the normalization method was used to analyze the differential expression of miRNAs. Fold change >2 with *p* value < 0.05 and fold change < 0.5 with *p* value < 0.05 were considered the default thresholds for significantly differential expression.

### Bioinformatics Analysis Based on Public Databases

The bioinformatics analysis based on TCGA database (https://genome‐cancer.ucsc.edu/) was first conducted to explore the expression of let‐7e‐5p and miR‐615‐3p in human BC tissues versus adjacent normal tissues. Then, Kaplan‐Meier plots for let‐7e‐5p expression and miR‐615‐3p expression in correlation with overall survival were generated based on TCGA database. Next, Pearson correlation analysis was performed based on TCGA data to evaluate the correlation of let‐7e‐5p or miR‐615‐3p expression with MICA or MICB expression. In addition, the potential binding sites in MICA or MICB mRNA of miRNAs were predicted using the mirWalk database and TargetScan database.

### Synthesis of Oxidized Starch (OS)

Soluble oxidized starch (OS) was obtained according to a previously published protocol.^[^ ^43^ ^]^ First, 50 mL of 10.56% (w/v) sodium periodate was added dropwise into 50 mL of starch aqueous solution, which was then placed in the 37 °C dark for three hours. Next, the oxidized products were precipitated with 150 mL of ethanol. Finally, FTIR spectroscopy (Thermos Scientific Nicolet IS10, USA) was used to evaluate the successful oxidization of starch.

### Synthesis of Mesoporous Bioactive Glass Nanoparticles (MBGNs)

Mesoporous bioactive glass nanoparticles (MBGNs) were produced using the method described in a previous study.^[^ ^33^ ^]^ First, 2.80 g hexadecyl trimethyl ammonium bromide was fully dissolved into 132 mL of deionized water, which was stirred at 35 °C. Subsequently, 40 mL ethyl acetate was put into hexadecyl trimethyl ammonium bromide solution. After the solution was stirred for 30 min, 28 mL ammonia solution (1 mol L^−1^) was added, and then the mixture was stirred for 15 min. Next, 14.40 mL tetraethyl orthosilicate was put into the mixture and stirred the mixture for 30 mins with a subsequent addition of 6.52 g calcium nitrate. After this step, the solution gradually became milky white, in which the colloids were formed after 4 h of stirring. Then, the colloidal particles were purified via centrifugation at 8000 rad s^−1^ and rinsed three times using water and three times with ethanol. These particles were further dried at 60 °C for 24 h and milled into fine powders by using a mortar. Finally, the powders were heated to 700 °C for 3 h at a heating rate of 2 °C min^−1^, which helped to remove the organics and nitrates that were mixed in the powders, consequently obtaining MBGNs.

### Drug Loading Capacity of MBGNs

To evaluate the drug loading capacity of MBGNs, SAHA or 3MA was first dissolved in PBS (pH 7.4). Then, MBGNs were added, and the mixture was stirred vigorously for 24 h at room temperature. Next, the mixture was centrifuged at 9000 rpm for 10 min to remove the supernatant and used 10 mL of deionized water to gently rinse the precipitate two times to clean the unloaded SAHA or 3MA. The drug concentrations of solutions were measured before and after the loading processes by Agilent HPLC system equipped with Agilent Zorbax SB‐C18 column (4.6 × 250 mm, Ø 5 µm). For detecting the concentrations of SAHA, the mobile phase A (HPLC grade methanol) and mobile phase B (DI‐H2O) were filtrated and degassed under a decreased pressure prior to usage. Chromatographic separation was run as a gradient elution (0 min: methanol 10%, 0–10 min: methanol 10–100%) at 37 °C. Through UV detection at 200 nm, SAHA was eluted at a retention time of 0.71 min and a flow rate of 1 mL min^−1^ within a total run time of 4 min. To detect the concentrations of 3MA, the mobile phase A (HPLC grade methanol) and mobile phase B (DI‐H2O) were filtrated and degassed under a decreased pressure prior to usage. Chromatographic separation was run as a gradient elution (0 min: methanol 10%, 0–10 min: methanol 10–100%) at 37 °C. Through UV detection at 220 nm, 3MA was eluted at a retention time of 0.82 min and a flow rate of 1 mL min^−1^ within a total run time of 4 min. In addition to HLPC, the fluorescein isothiocyanate (FITC)‐labeled drug model was also used to evaluate the drug loading capacity of MBGNs as previously described.^[^ ^55^, ^56^ ^]^ First, SAHA and 3MA were first labeled with FITC. In a brief, SAHA or 3MA, FITC, and PBS were mixed in a 5 mL round‐bottom flask, and the reaction mixture was stirred at room temperature in pH = 9.0 PBS at the dark condition for 8 h. The product was transferred into dialysis cellulose tubing (Sigma Aldrich, St Louis, MO, USA) using a typical molecular weight cutoff of 14 000 Da to eliminate the unbound FITC. Dialysis was carried out for 7 d against ultrapurified water, protected from light, at room temperature, and the water was changed every day. The content of the dialysis membrane was subsequently transferred into a falcon tube and freeze‐dried (protected from light) to obtain FITC‐labeled SAHA or 3MA. Then, FITC‐labeled SAHA or 3MA was dissolved in PBS (pH 7.4). Next, MBGNs were added, and the mixture was stirred vigorously for 24 h at room temperature, which was centrifuged at 9000 rpm for 10 min to remove the supernatant and used 10 mL of deionized water to gently rinse the precipitate two times to clean the unloaded SAHA or 3MA. The solutions were detected before and after the loading processes via a UV–vis spectrometer (Lambda 35, PerkinElmer) at a wavelength of 460 nm to quantify the loaded drugs. The drug loading capacity was calculated using the formula loading capacity = (M0‐M1)/MS. Herein, M0 stands for the total mass of SAHA or 3MA, M1 represents the mass of SAHA or 3MA in the supernatant after the drug loading procedures, and MS represents the mass of MBGNs.

### Fabrication of Gel‐OS/SAHA@3MA@MBGN Hydrogels

To fabricate the Gel‐OS/SAHA@3MA@MBGN hydrogel (GOSAM), the oxidized starch (OS) was first dispersed into an aqueous solution to make an OS solution (10% w/v). Then, SAHA and 3MA‐loaded MBGNs (5% w/v) were added to the OS solution, which was subjected to ultrasonic dispersion for 10 min at room temperature, ultimately obtaining the MBGN‐containing OS solution. In addition, gelatin was dissolved in aqueous solution (0.05 m) at 60 °C to make the gelatin solution (30%, w/v). Finally, the MBGN‐containing OS solution and the gelatin solution were fully mixed, and subsequently, the mixture was poured into a mold to fabricate GOSAM at 37 °C.

### Rheological Test

Rheological tests were performed with reference to published studies.^[^ ^45^ ^]^ The linear viscosity (*η*) under the frequency sweep mode was measured to evaluate the injectability of the hydrogel. The strain sweep measurements were implemented over a range from 0.1% to 1000% strain. The wet tissue adhesion of the hydrogel was evaluated using a previous protocol. Briefly, the hydrogel precursor solution was injected uniformly between two pieces of porcine skins (10 mm × width and 20 mm length), in which the hydrogel‐crosslinked interface between the two pieces of pig skins was 1 cm^2^. After 20 min, the lap‐shear test was conducted using a dynamic mechanical analyzer (DMA Q800, USA) at a speed of 1 mm min^−1^.

### Biocompatibility and Biodegradation of the Gel‐OS/SAHA@3MA@MBGN Hydrogel

To evaluate the biocompatibility of the Gel‐OS/SAHA@3MA@MBGN hydrogel (GOSAM), blood compatibility assays were first conducted using the protocol reported by Liang et al.^[^ ^32^ ^]^ In brief, 100 µL GOSAM, PBS or 1% Triton X‐100 was added to 500 µL blood pretreated with sodium citrate anticoagulant, and subsequently, the blood was placed in a shaker at 37 °C for 3 h. Next, the blood was centrifuged at 4000 rpm for 10 min and gathered 200 µL supernatant, into which 5 mL deionized water was then added. Finally, UV–vis spectroscopy was utilized to measure the absorbance at 540 nm and then calculated the hemolysis ratio based on the absorbance data. In addition, cell viability and live/dead staining assays were conducted to further assess the biocompatibility of the hydrogel. Briefly, 100 µL hydrogel was first added onto the bottom of 96‐well plates, incubated these plates for 4 h and then washed the plates three times with PBS. Next, human normal mammary epithelial cells (MCF‐10A) were planted on the surfaces of the hydrogel (100 µL per well, 6000 cells per well) and incubated at 5% CO_2_ and 37 °C for 24, 48, or 72 h. After incubation, a CCK‐8 kit was used to detect cell viability. The live/dead cell staining assays were carried out in six‐well plates (300 µL per well, 50 000 cells per well). MCF‐10A cells planted on the surfaces of the hydrogel were first stained according to the live/dead cell double staining kit and then observed by an inverted fluorescence microscope (Olympus, BX51, Japan). To evaluate the in vivo compatibility, the hydrogel was first injected into mice subcutaneously and then sampled blood from mice 3, 10, and 21 d after the hydrogel injection, which was subjected to biochemical tests. To assess biodegradation, the hydrogel was first soaked in 5 mL pH 7.4 and pH 6.5 PBS solution at 37 °C, which was then shaken at a constant speed of 90.0 rpm. Next, the residual hydrogels were rinsed using PBS solution and weighed them under wet conditions. The in vivo degradation ability of the hydrogel was evaluated as previously described.^[^ ^57^, ^58^ ^]^ Briefly, gelatin with Cy5.5 was first labeled and then fabricated the hydrogel using the Cy5.5‐labeled gelatin. Then, the hydrogel was injected into mice subcutaneously, and its degradation process was monitored via the Cy5.5 signal detected by the fluorescence in vivo imaging system. In addition, the general observation of the hydrogels 1, 7, 14, and 21 d was also made after they were subcutaneously injected into mice, so as to further evaluate the degradation behavior of the hydrogels.

### Drug Release of the Gel‐OS/SAHA@3MA@MBGN Hydrogel

To investigate the drug release behavior in vitro, the hydrogel was soaked in pH 7.4 or pH 6.5 PBS solution, which was softly shaken at 37 °C. The supernatant was collected and changed at the selected time points. The released drug in the supernatant was quantitatively assessed by Agilent HPLC system equipped with Agilent Zorbax SB‐C18 column (4.6 × 250 mm, Ø 5 µm). For evaluating the release of SAHA, the mobile phase A (HPLC grade methanol) and mobile phase B (DI‐H2O) were filtrated and degassed under a decreased pressure prior to usage. Chromatographic separation was run as a gradient elution (0 min: methanol 10%, 0–10 min: methanol 10–100%) at 37 °C. Through UV detection at 200 nm, SAHA was eluted at a retention time of 0.71 min and a flow rate of 1 mL min^−1^ within a total run time of 4 min. For assessing the release of 3MA, the mobile phase A (HPLC grade methanol) and mobile phase B (DI‐H2O) were filtrated and degassed under a decreased pressure prior to usage. Chromatographic separation was run as a gradient elution (0 min: methanol 10%, 0–10 min: methanol 10–100%) at 37 °C. Through UV detection at 220 nm, 3MA was eluted at a retention time of 0.82 min and a flow rate of 1 mL min^−1^ within a total run time of 4 min. In addition to HPLC, FITC‐labeled drug model was also adopted to further evaluate the drug release behavior in vitro. In this experiment, FITC‐labeled SAHA or 3MA was used to fabricate the hydrogels. Then, the hydrogels were soaked in pH 7.4 or pH 6.5 PBS solution, which was softly shaken at 37 °C. The supernatant was collected and changed at the selected time points. The released drug in the supernatant was quantitatively assessed via the UV–Vis–NIR absorbance spectrum. The drug release in vivo was evaluated as previously described.^[^ ^57^, ^58^ ^]^ Briefly, SAHA and 3MA were first labeled with Cy5.5 and used to fabricate the hydrogels. Then, Cy5.5‐marked hydrogels were injected into the tumor resection bed of a triple‐negative breast cancer (TNBC) mouse model. After injection, the drug release behavior was monitored by detecting Cy5.5 signal in the residual hydrogel with the help of in vivo imaging system.

### Isolation and Expansion of Umbilical Cord Blood NK Cells

Human umbilical cord blood (UCB) samples were acquired from the Third Affiliated Hospital of Sun Yat‐Sen University based on the protocol, which was approved by the Ethical Committee department (No. [2021]02‐226‐01). Written informed consent was also obtained from the UCB donor. The isolation and expansion of UCB‐NK cells were performed via the IL‐21 natural killer cell amplification system purchased from Hangzhou Zhongying Technology Co., Ltd. (Hangzhou, China).

### NK Cell Cytotoxicity Assay

Target ALDH1+ breast cancer stem cells (BCSCs) or ALDH1‐ breast cancer cells were plated at 1 × 10^4^ cells per well. When target cells adhered to the bottom of plates, human umbilical cord NK cells at a 1:1 ratio were added to target cells incubated in a 96‐well plate in the presence of SAHA and/or 3MA. Then, the xCELLigence real‐time cell analysis (RTCA) Instrument system was utilized to evaluate NK cell cytotoxicity to target cells by detecting the cell index. In addition, NK cell cytotoxicity was assessed by flow cytometry as previously reported.

### Establishment of an Orthotopic CSC‐Enriched TNBC Mouse Model

To establish a CSC‐enriched TNBC mouse model, ALDH1+ breast cancer stem cells (BCSCs) were first isolated from the human TNBC cell line MDA‐MB‐231 as previously reported.^[^ ^4^ ^]^ Next, BCSCs were transfected with recombinant lentiviruses expressing firefly luciferase and mCherry, which helps to isolate BCSCs from the tumor tissues of mice for further analysis. Then, these transfected BCSCs (5 × 10^5^) were injected into the breast pad of NOD‐SCID IL‐2 receptor gamma null (NSG) mice to establish an orthotopic CSC‐enriched TNBC model.

### In Vivo Inhibition of Tumor Growth and Relapse

To explore the in vivo effect of hydrogels on tumor growth in the orthotopic CSC‐enriched TNBC mouse model, TNBC‐bearing mice with ≈200 mm^3^ tumors were treated with PBS, UCB‐NK‐cell infusion (5 × 10^6^), NK cell infusion combined with drug‐unloaded hydrogel intratumor injection, NK cell infusion with SAHA‐loaded (55 mg kg^−1^) hydrogel intratumor injection, NK cell infusion with 3MA‐loaded (35 mg kg^−1^) hydrogel intratumor injection, NK cell infusion with intratumor injection of hydrogel encapsulating SAHA and 3MA, or NK cell infusion with intravenous injection of SAHA (15 mg kg^−1^) and 3MA (10 mg kg^−1^). Tumor progression was monitored by an in vivo bioluminescence imaging system, and the luminescence intensity was used to quantitatively evaluate tumor growth as previously reported.^[^ ^57^ ^]^ To investigate the effect of hydrogels on tumor relapse, tumor resection was first performed on TNBC‐bearing mice and then randomly allocated mice into seven groups, which were treated with PBS, UCB‐NK infusion alone (5 × 10^6^), NK infusion with drug‐unloaded hydrogel injection onto the tumor resection bed, NK cell infusion with SAHA‐loaded (55 mg kg^−1^) hydrogel injection, NK cell infusion with 3MA‐loaded (35 mg kg^−1^) hydrogel injection, NK cell infusion with injection of hydrogel encapsulating SAHA and 3MA, or NK cell infusion with intravenous injection of SAHA (15 mg kg^−1^) and 3MA (10 mg kg^−1^). Tumor relapse and regrowth were also monitored using an in vivo bioluminescence imaging system.

### In Vivo Hemostatic Effect Testing

The in vivo hemostatic function of the hydrogel was investigated in multiple rat bleeding models, including the tail amputation model, liver slice resection wound bleeding model and liver partial resection bleeding model, as previously described.^[^ ^32^ ^]^ Each animal was euthanized using CO_2_ inhalation when the experiment was finished. The hemostatic experiments were approved by the Animal Care and Use Committee of Sun Yat‐Sen University (No. IACUC‐ F3‐22‐0207).

### Wound Healing Assessment

Female BALB/c mice were used for wound healing assessment and were randomly allocated into four groups before surgery. After anesthetization by intraperitoneal injection of pentobarbital sodium, mice were given a round full‐thickness skin wound on the back. Among these groups, three groups of mice were treated with drug‐unloaded hydrogel, 3MA‐loaded hydrogel and SAHA‐3MA‐loaded hydrogel. Additionally, one group of mice was treated with gauze, which was set as the control group. The wound area was observed and measured at the designated time points. Mice were euthanized at the indicated times (5, 10, and 20 d after implantation), and the regenerated skin tissues were sampled for H&E and Masson staining experiments.

### Statistical Analysis

The relative miRNA levels were normalized to RNU6B according to the 2^−ΔΔct^ method before statistical comparison. All data were presented as the mean ± SD. The sample size in each group was 5 and 12 for statistical analysis in animal experiments about tumor growth and tumor relapse, respectively. The sample size in each group was 4 in the experiments of HE staining and Masson staining. The sample size (*n* = 3) was for the other experiments. Student's t test or Wilcoxon rank‐sum test were used to compare the difference between two groups. Kaplan‐Meier survival analysis with log rank test was performed to determine overall survival (OS). The statistical difference was defined by *p < 0.05 as significant, **p < 0.01 as moderately significant, and ***p < 0.001 as highly significant, respectively. All statistical analyses were conducted using SPSS, version 24 (IBM).

## Conflict of Interest

The authors declare no conflict of interest.

## Supporting information

Supporting Information

Supplemental Table 1

Supplemental Table 2

## Data Availability

The data that support the findings of this study are available in the supplementary material of this article.
