# Supplementary material for: An Injectable Epigenetic Autophagic Modulatory Hydrogel for Boosting Umbilical Cord Blood NK Cell Therapy Prevents Postsurgical Relapse of Triple‐Negative Breast Cancer
Source: Adv Sci (Weinh). 2022 Jun 16;9(23):2201271. doi: 10.1002/advs.202201271 (PMC9376812; doi:10.1002/advs.202201271)
Supplement: Supplementary file 1 — Supporting Information [file ADVS-9-2201271-s003.pdf]

ADVANCED SCIENCE
Open Access

Supporting Information

for *Adv. Sci.*, DOI 10.1002/advs.202201271

An Injectable Epigenetic Autophagic Modulatory Hydrogel for Boosting Umbilical Cord Blood NK Cell Therapy Prevents Postsurgical Relapse of Triple-Negative Breast Cancer

Yihang Gong, Wenjie Chen, Xiuxing Chen, Yizhan He, Hua Jiang, Xijian Zhang, Lijie Pan, Beibei Ni, Fan Yang, Yan Xu, Qi Zhang, Lei Zhou* and Yusheng Cheng**

Copyright WILEY-VCH Verlag GmbH & Co. KGaA, 69469 Weinheim, Germany, 2018.

Supporting Information

An Injectable Epigenetic Autophagic Modulatory Hydrogel for Boosting Umbilical Cord Blood NK Cell Therapy Prevents Postsurgical Relapse of Triple-Negative Breast Cancer

Yihang Gong, Wenjie Chen, Xiuxing Chen, Yizhan He, Hua Jiang, Xijian Zhang, Lijie Pan, Beibei Ni, Fan Yang, Yan Xu, Qi Zhang, Lei Zhou* and Yusheng Cheng**

Dr. Y. Gong, Dr. Y. Cheng, Dr. X. Zhang
Department of Hepatic Surgery and Liver Transplantation Center & Guangdong Provincial Key Laboratory of Liver Disease Research
The Third Affiliated Hospital, Sun Yat-sen University
Guangzhou, 510630, China
E-mail: chengyusheng2017@163.com

Prof. Q. Zhang, Y. He, L. Pan, B. Ni, F. Yang, Y. Xu, and Prof. W. Chen
Biotherapy Centre & Cell-gene Therapy Translational Medicine Research Centre
The Third Affiliated Hospital, Sun Yat-sen University
Guangzhou, 510630, China
E-mail: zhangq27@mail.sysu.edu.cn

X. Chen
Guangdong Provincial Key Laboratory of Malignant Tumor Epigenetics and Gene Regulation, Department of Medical Oncology
Sun Yat-sen Memorial Hospital, Sun Yat-sen University
Guangzhou, 510120, China

Dr. H. Jiang
Department of Breast & Thyroid Surgery
The Third Affiliated Hospital, Sun Yat-sen University
Guangzhou, 510630, China

Dr. L. Zhou
Guangzhou Key Laboratory of Spine Disease Prevention and Treatment, Department of Spine Surgery

The Third Affiliated Hospital of Guangzhou Medical University
Guangzhou, 510150, China
E-mail: zhoul@gzhmu.edu.cn

Keywords: Hydrogel, Sustained drug release, Wound healing, UCB-NK cell therapy, TNBC

Figure S1

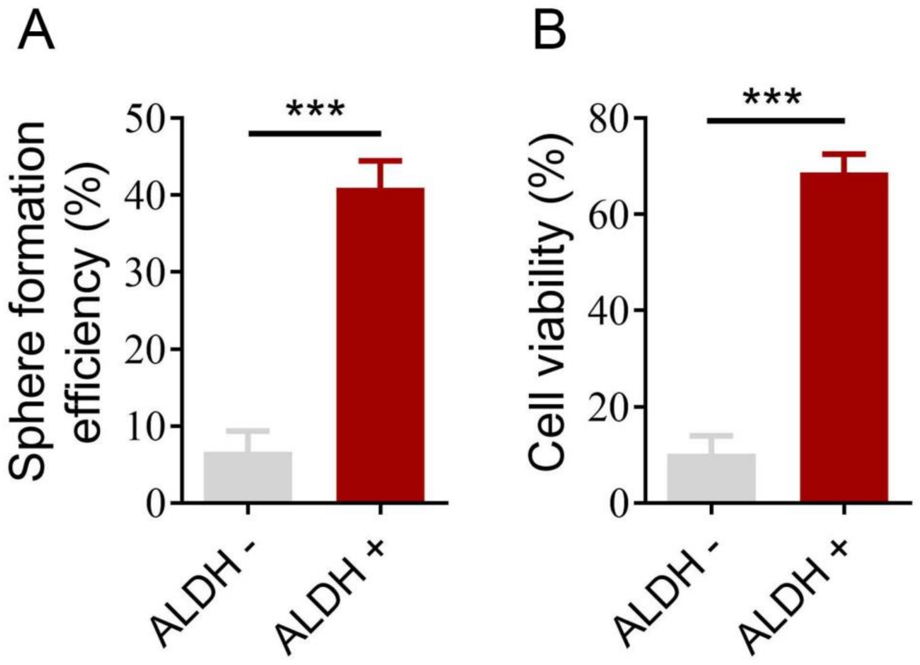

Figure S1. A) Quantification of the sphere formation efficiency of ALDH- and ALDH+ BCSCs; B) Quantification of lysis effect of UCB-NK cells on ALDH- and ALDH+ BCSCs. Data are presented as mean ± SD, (n = 3). ***p < 0.001.

Figure S2

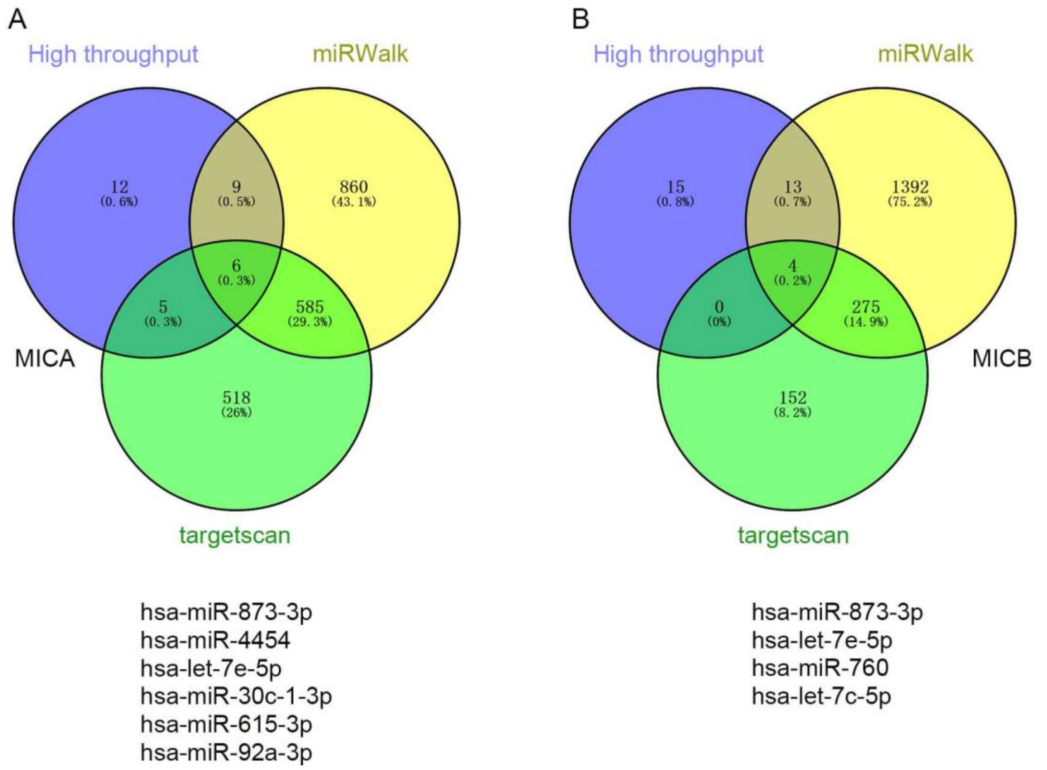

Figure S2. A) Different miRNAs predicted by combining high-throughput sequencing and mirWalk and targetscan databases can bind to MICA; B) Different miRNAs predicted by combining high-throughput sequencing and mirWalk and targetscan databases can bind to MICB.

Figure S3

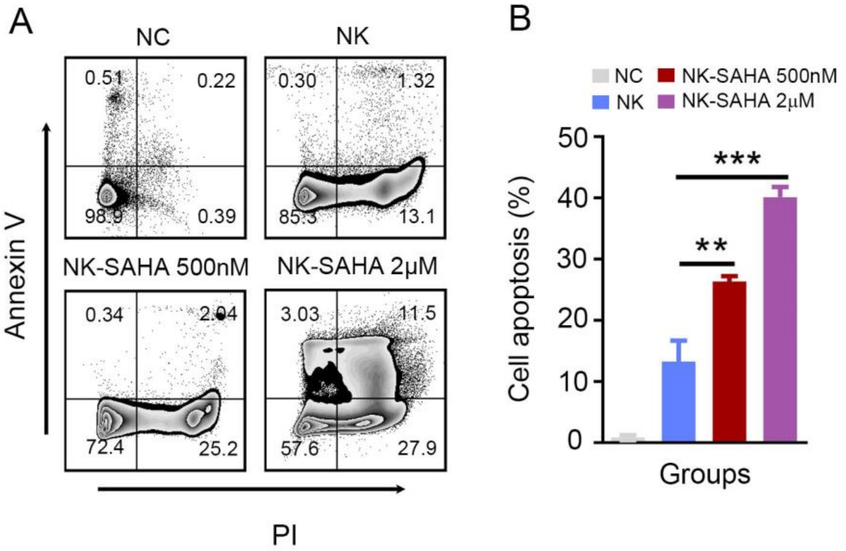

Figure S3. A) The apoptosis of different groups analyzed by flow cytometry; B) Quantification of apoptosis of different groups. Data are presented as mean ± SD, (n = 3). $^{**}p < 0.01$ and $^{***}p < 0.001$

Figure S4

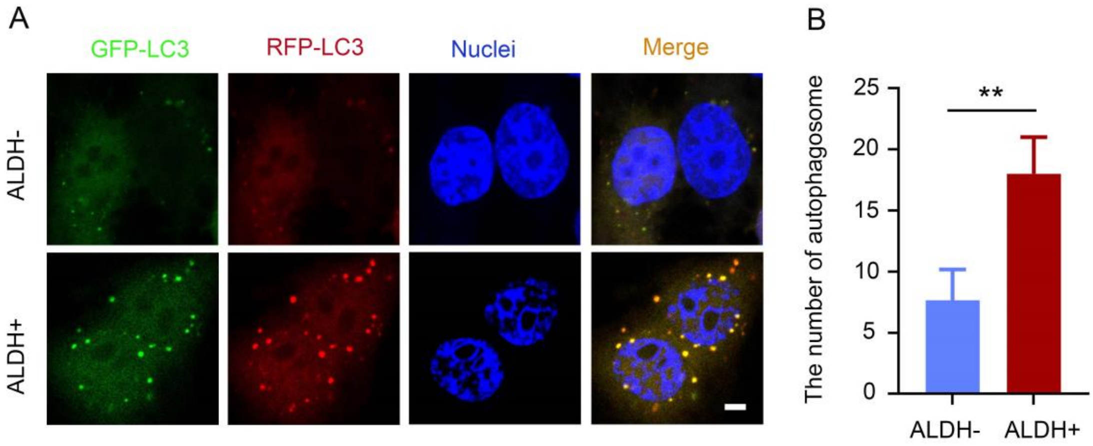

Figure S4. A) The autophagy level measured by Immunofluorescence staining of ALDH1- and ALDH1+ BCSCs; B) Quantification of autophagy level of ALDH1- and ALDH1+ BCSCs. Data are presented as mean ± SD, (n = 3). **p < 0.01. Scale bar=2μm.

Figure S5

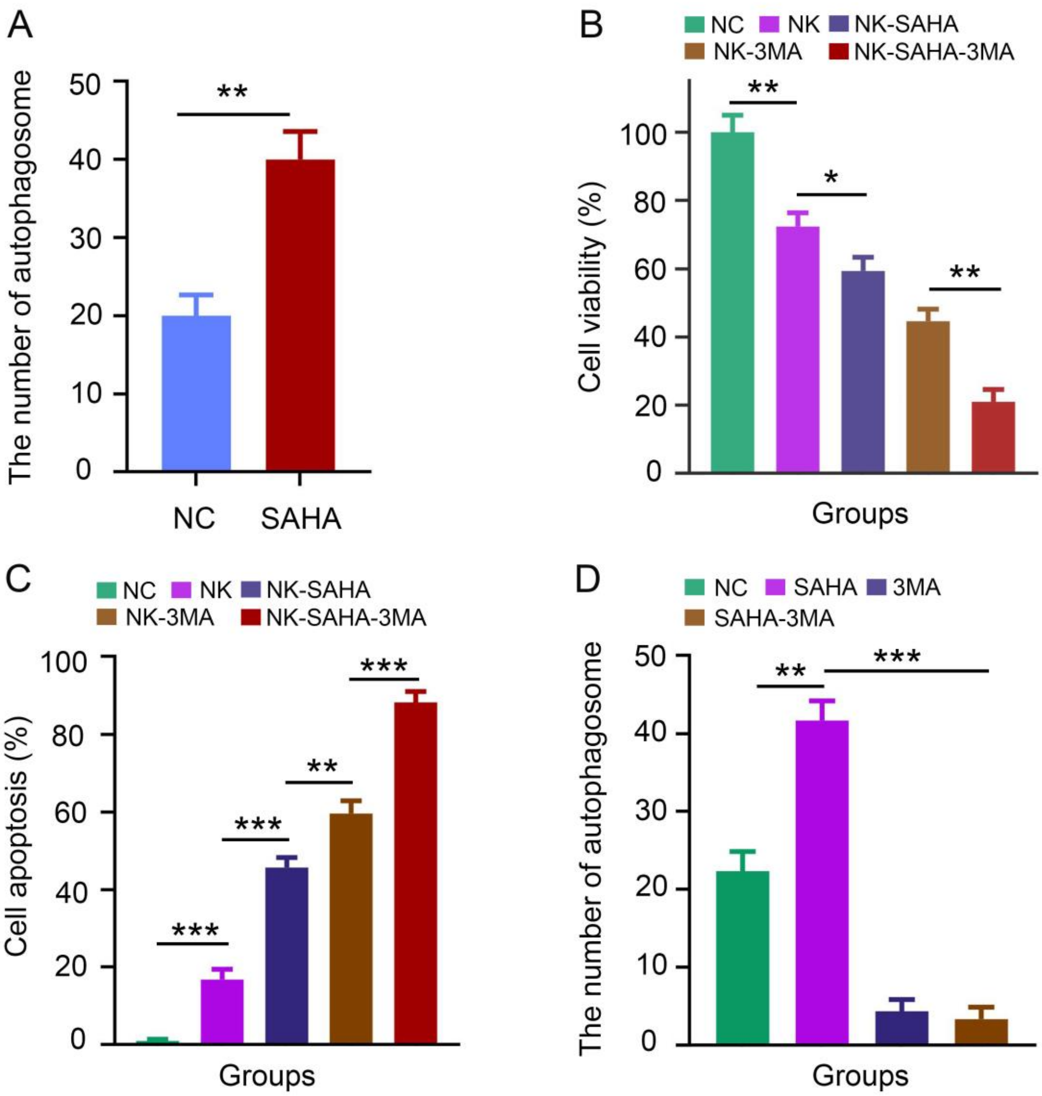

Figure S5. A) Quantification of autophagy level in different groups; B) Quantification of lytic capacity of UCB-NK cells under different treatment C) The apoptosis of ALDH+ BCSCs treated with different methods. D) Quantification of the effect of SAHA and 3MA on autophagy levels in ALDH+ BCSCs. Data are presented as mean ± SD, (n = 3). *p < 0.05, **p < 0.01, and ***p < 0.001.

Figure S6

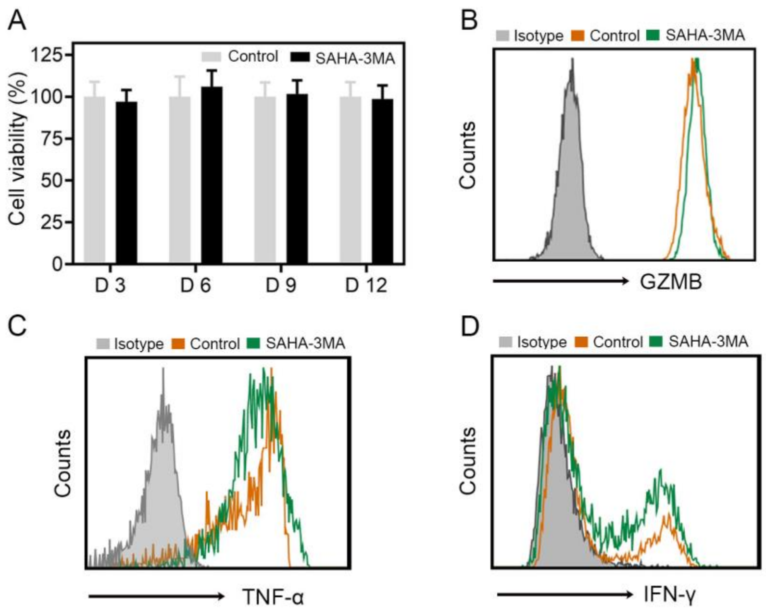

Figure S6. A) The cell viability of different groups measured by CCK8 assay; B) The expression of GZMB analyzed by flow cytometry in the presence or absence of SAHA and 3MA; C-D) The expression of TNF-α and IFN-γ analyzed by flow cytometry in the presence or absence of SAHA and 3MA. Data are presented as mean ± SD, ($n = 3$). *$p < 0.05$, **$p < 0.01$, and ***$p < 0.001$.

Figure S7

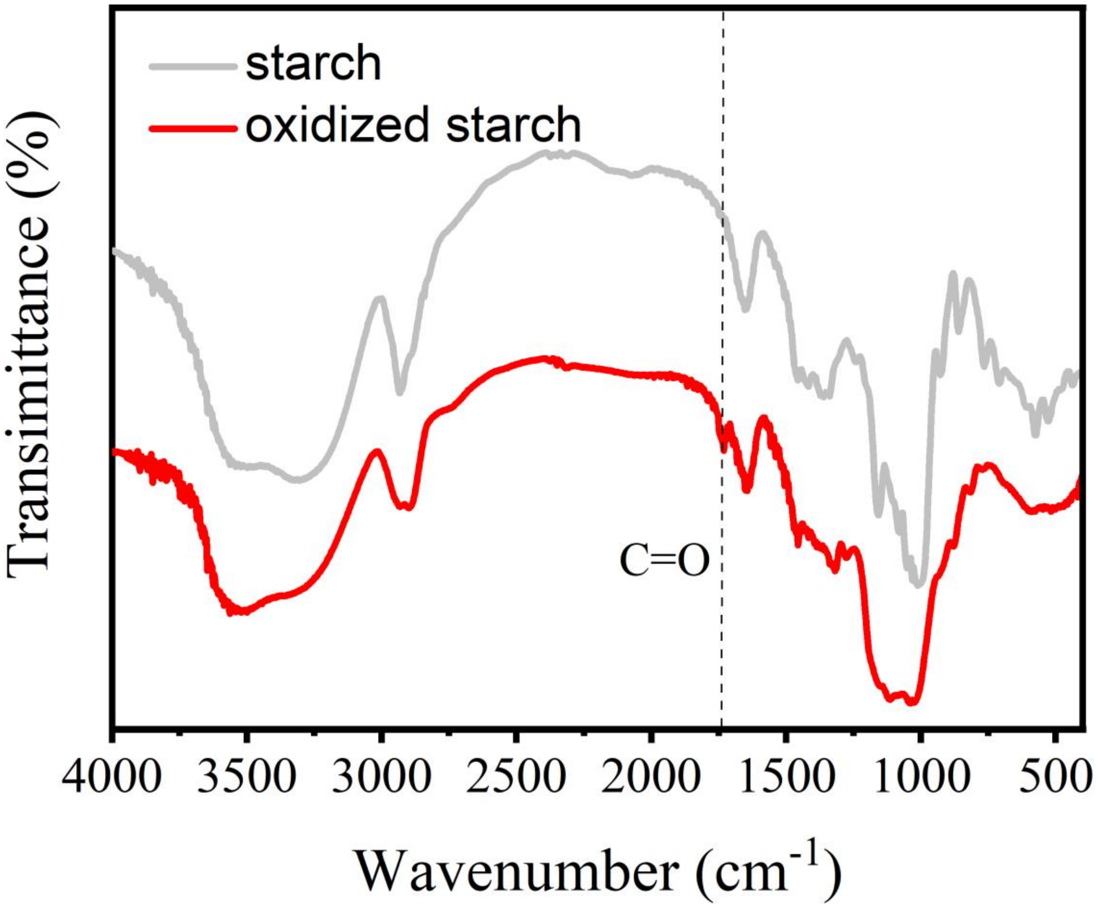

Figure S7. Fourier transform infrared (FTIR) spectra of starch and oxidized starch (OS).

Figure S8

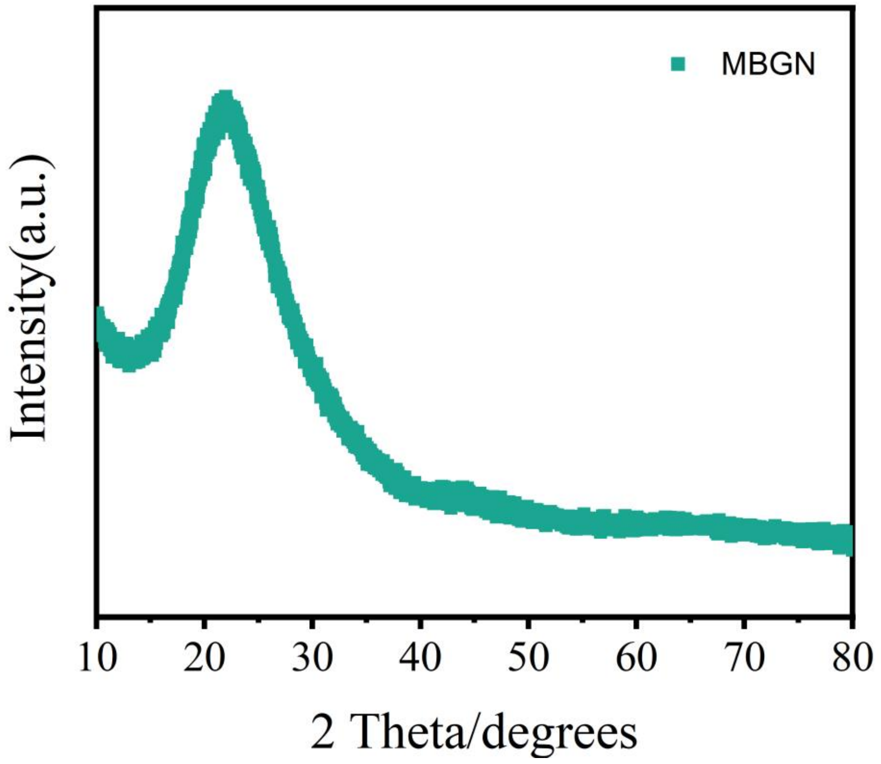

Figure S8. X-ray diffraction (XRD) pattern of MBGNs.

Figure S9

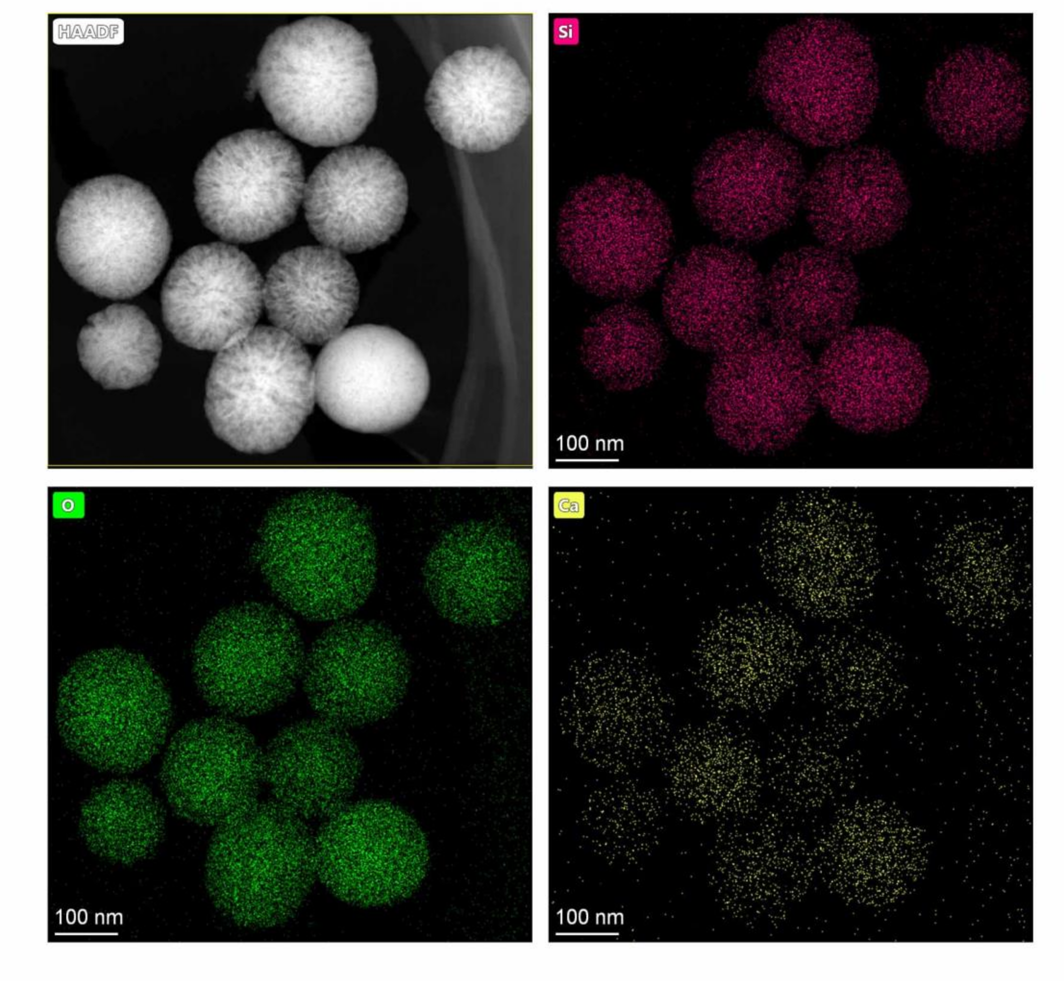

Figure S9. Scanning transmission electron microscope (TEM) images of MBGNs showing the elements of Si (green), Ca (red) and oxygen (green). Scale bar = 100 nm.

Figure S10

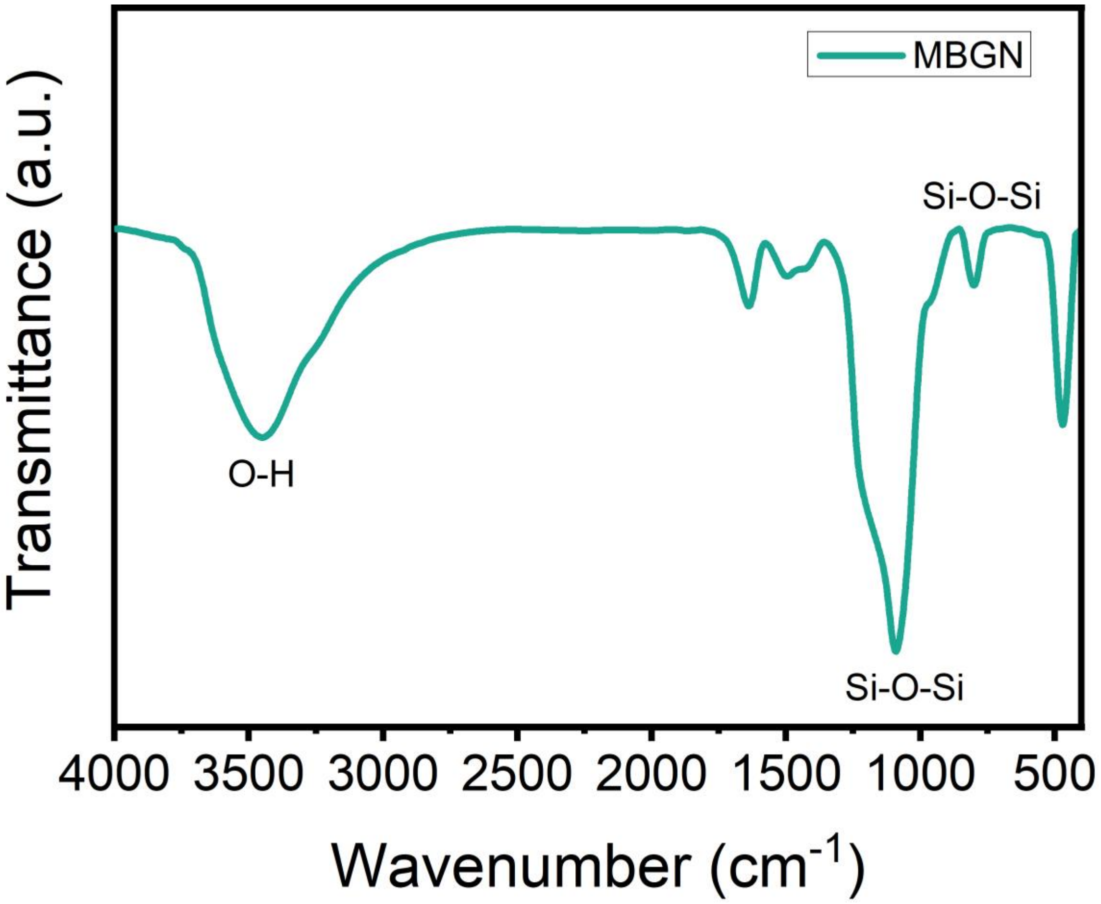

Figure S10. FTIR spectra of MBGNs.

Figure S11

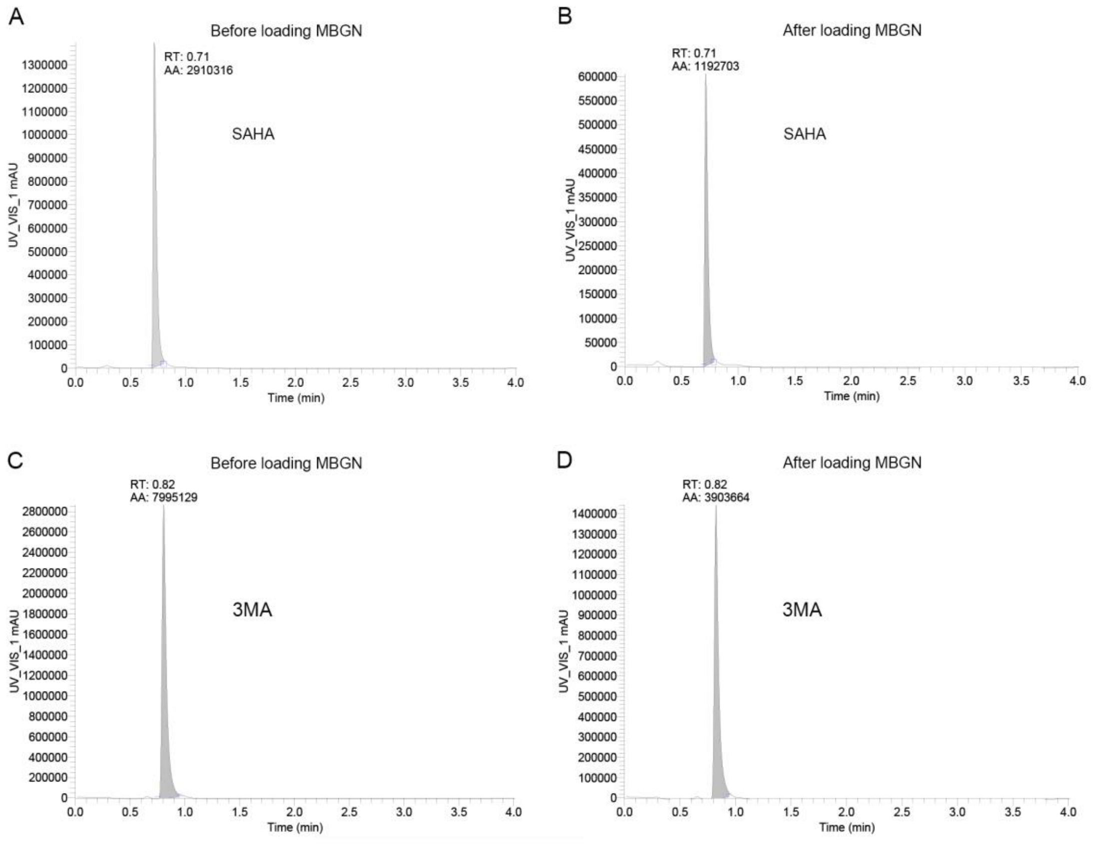

Figure S11. A-B) The SAHA loading capacity of MBGNs. **C-D**) The 3MA loading capacity of MBGNs.

Figure S12

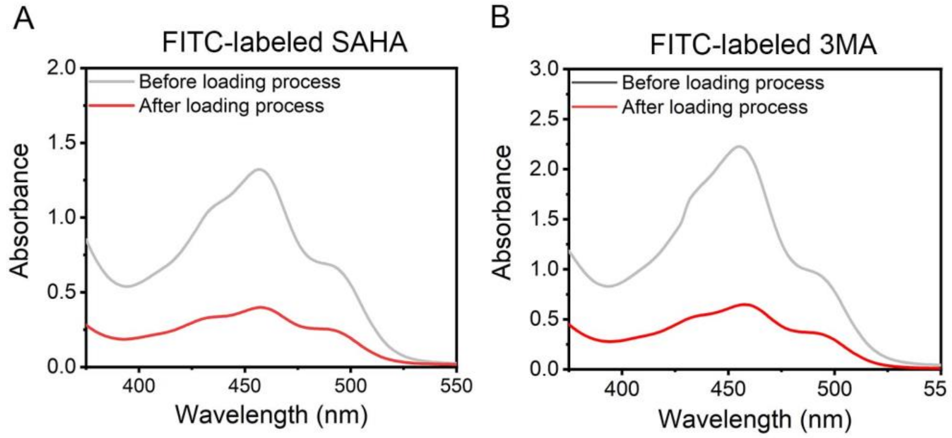

Figure S12. A) The FITC-labeled SAHA loading capacity of MBGNs. **B**) The FITC-labeled 3MA loading capacity of MBGNs.

Figure S13

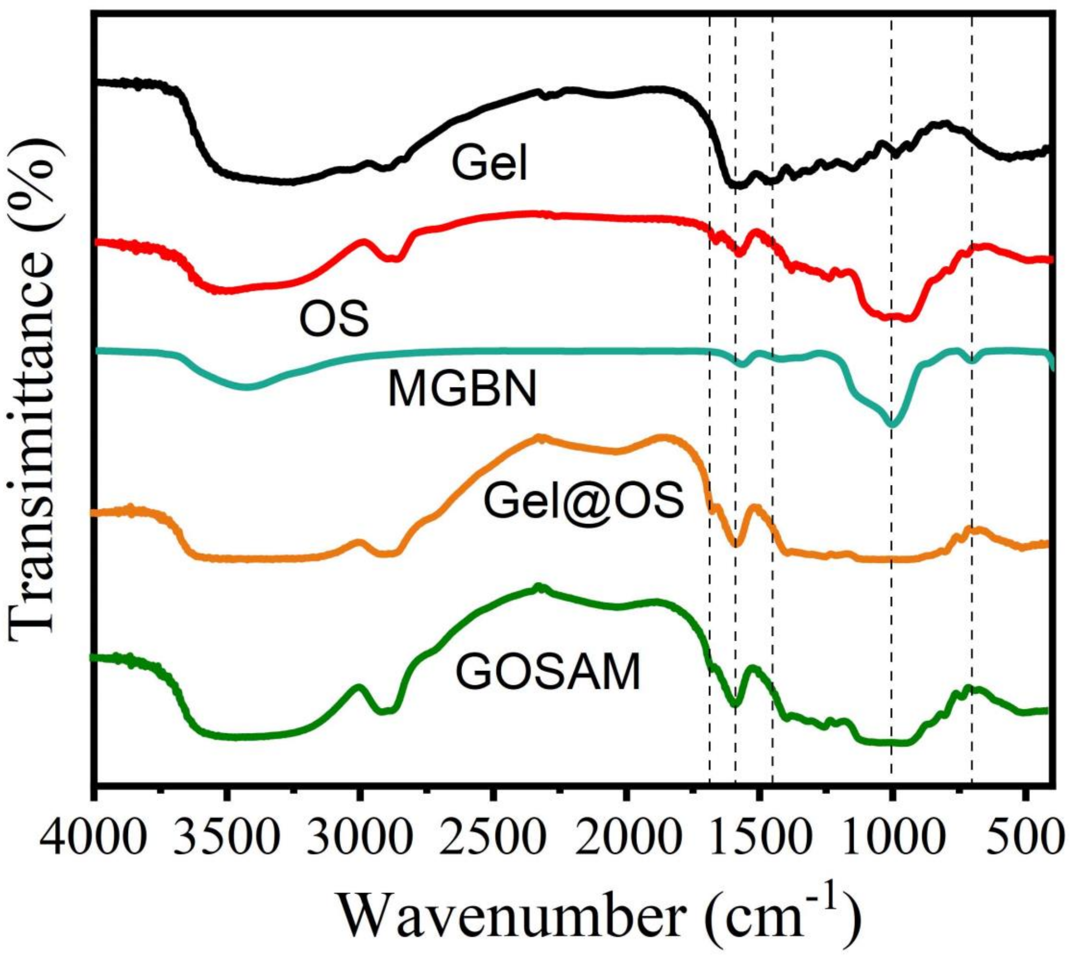

Figure S13. FTIR spectra of Gel-OS/SAHA@3MA@MBGNs Hydrogel (GOSAM).

Figure S14

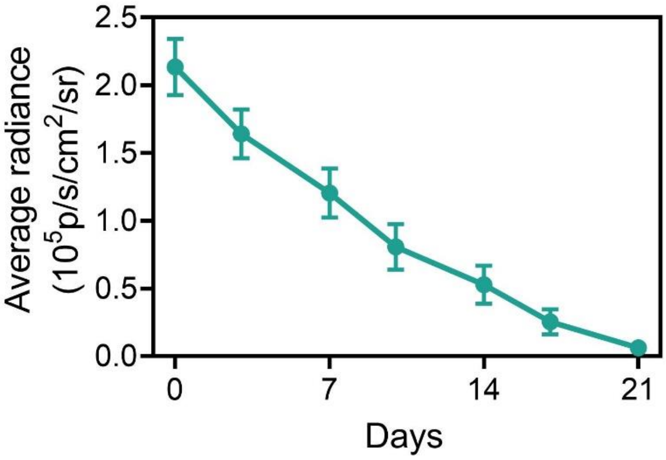

Figure S14. The quantitative results of fluorescence signals of Cy5.5-labeled GOSAM in (Figure 6F). Data are presented as the mean ± SD, (n = 3).

Figure S15

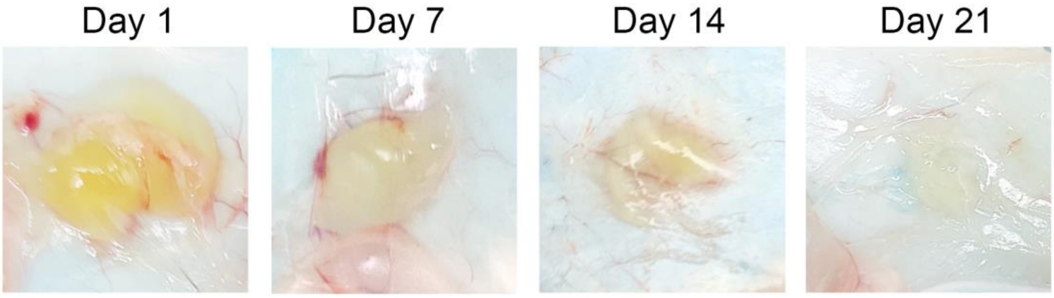

Figure S15. The general observation of GOSAM subcutaneously injected into mice.

Figure S16

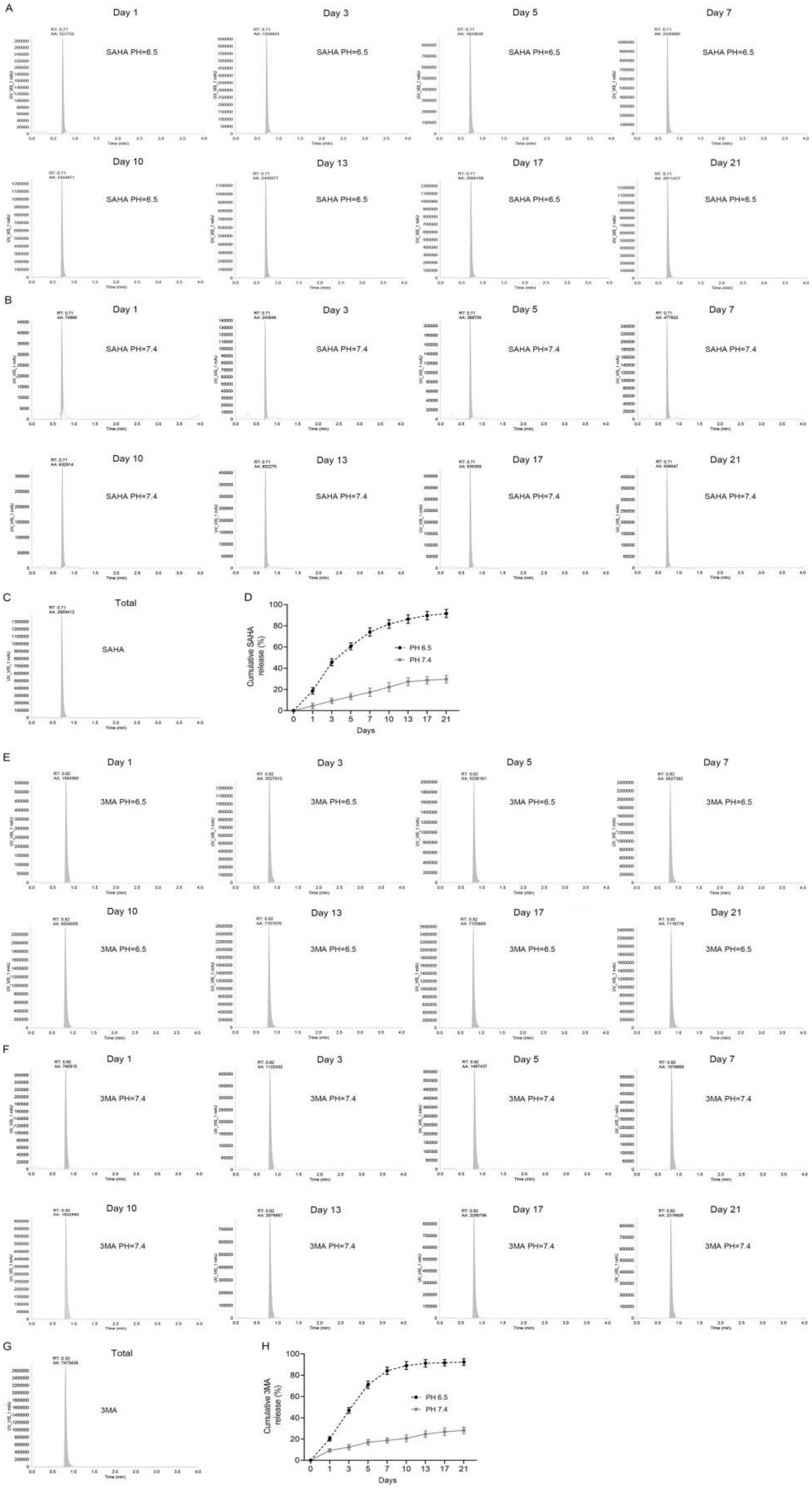

Figure S16. A) The concentrations of SAHA from GOSAM in pH 6.5 PBS at multiple time points;

B) The concentrations of SAHA from GOSAM in pH 7.4 PBS at multiple time points; C) SAHA in ethanol with retention time of 0.71 min as detected by UV detector at 200 nm. D) Accumulative release curves of SAHA from GOSAM in pH 6.5 PBS and 7.4 PBS. Data are presented as the mean ± SD, (n = 3); E) The concentrations of 3MA from GOSAM in pH 6.5 PBS at multiple time points; F) The concentrations of 3MA from GOSAM in pH 7.4 PBS at multiple time points; G) 3MA in ethanol with retention time of 0.82 min as detected by UV detector at 220 nm. H) Accumulative release curves of SAHA from GOSAM in pH 6.5 PBS and 7.4 PBS. Data are presented as the mean ± SD, (n = 3).

Figure S17

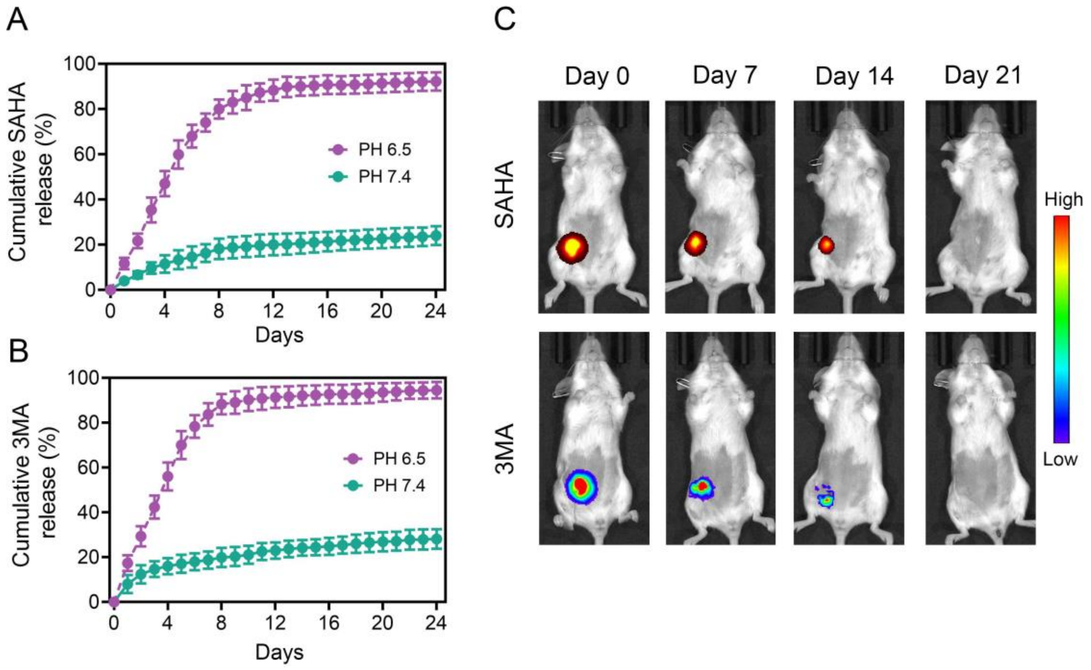

Figure S17. A) Accumulative release curves of FITC-labeled SAHA from GOSAM in pH 6.5 PBS and 7.4 PBS; B) Accumulative release curves of FITC-labeled SAHA from GOSAM in pH 6.5 PBS and 7.4 PBS; C) Fluorescence IVIS imaging monitoring the in vivo retention of Cy5.5-labelled SAHA or 3MA encapsulated in GOSAM injected into the breast pad. Data are presented as the mean ± SD, (n = 3).

Figure S18

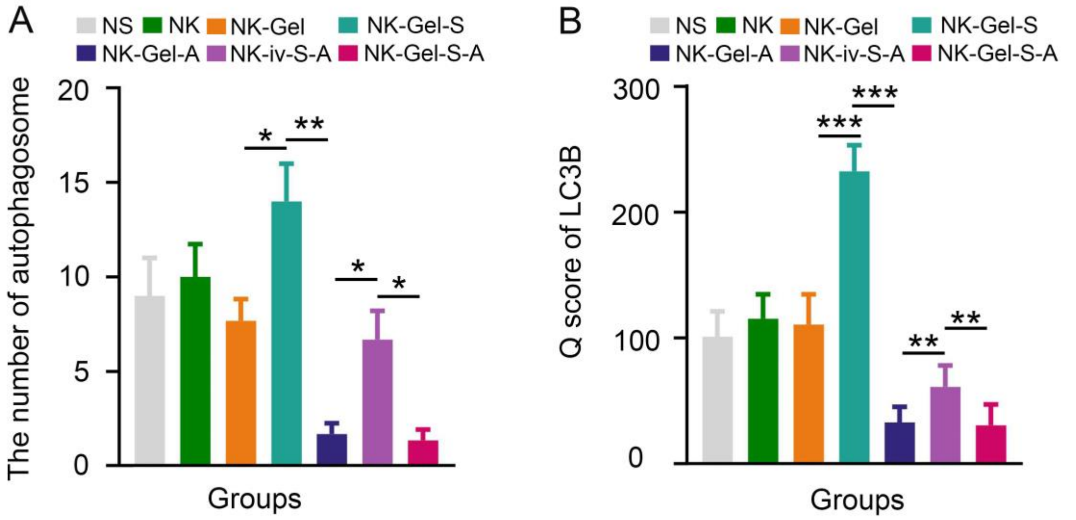

Figure S18. A) Quantification of autophagosome in different groups; B) Quantification of LC3B in different treatment. Data are presented as mean ± SD, (n = 5). *p < 0.05, **p < 0.01, and ***p < 0.001.

Figure S19

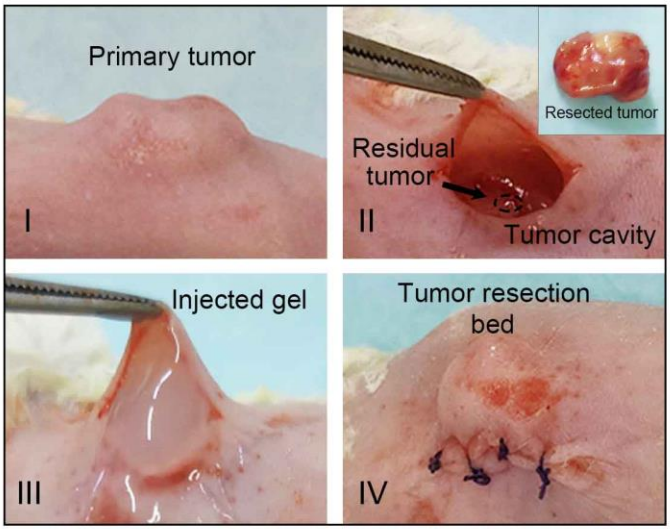

Figure S19. Resection and injection approach: (I) Surgery was performed after the tumor volume reached ≈200 mm^3. (II) Incomplete tumor resection model was simulated (approximately 95 % of primary tumor was excised) (III) Injection of the GOSAM-gel. (IV) Wound closure.
